# Transformer for one stop interpretable cell type annotation

**DOI:** 10.1038/s41467-023-35923-4

**Published:** 2023-01-14

**Authors:** Jiawei Chen, Hao Xu, Wanyu Tao, Zhaoxiong Chen, Yuxuan Zhao, Jing-Dong J. Han

**Affiliations:** Peking-Tsinghua Center for Life Sciences, Academy for Advanced Interdisciplinary Studies, Center for Quantitative Biology (CQB), Peking University, Beijing, 100871 China

**Keywords:** Machine learning, Software

## Abstract

Consistent annotation transfer from reference dataset to query dataset is fundamental to the development and reproducibility of single-cell research. Compared with traditional annotation methods, deep learning based methods are faster and more automated. A series of useful single cell analysis tools based on autoencoder architecture have been developed but these struggle to strike a balance between depth and interpretability. Here, we present TOSICA, a multi-head self-attention deep learning model based on Transformer that enables interpretable cell type annotation using biologically understandable entities, such as pathways or regulons. We show that TOSICA achieves fast and accurate one-stop annotation and batch-insensitive integration while providing biologically interpretable insights for understanding cellular behavior during development and disease progressions. We demonstrate TOSICA’s advantages by applying it to scRNA-seq data of tumor-infiltrating immune cells, and CD14+ monocytes in COVID-19 to reveal rare cell types, heterogeneity and dynamic trajectories associated with disease progression and severity.

## Abstract (web summary)

Developing computational tools for interpretable cell type annotation in scRNA-seq data remains challenging. Here the authors propose a Transformer-based model for interpretable annotation transfer using biologically understandable entities, and demonstrate its performance on large or atlas datasets.

## Introduction

Single-cell technologies have enabled studying biological processes and human diseases at unprecedented resolution and transformed the tool boxes in biology. An important step in scRNA-seq analysis is to identify cell populations or types by clustering^1^. Cell type annotation can resolve cellular heterogeneity across tissues, developmental stages and organisms, and improve our understanding of cellular and gene functions in health and disease. Many unsupervised scRNA-seq clustering methods have been proposed^2–4^, which are followed by time-consuming and labor-costly annotations^5^. These traditional methods often consist of preprocessing, dimensionality reduction, clustering, differential analysis, and manual annotation based on prior knowledge. When subtypes are annotated manually based on a small set of marker genes, the same subtype can sometimes be given a new name in another research due to a slight difference. Also, when all samples cannot be obtained at the same time, it would be desirable to classify the cell types on the first batch of data and use them to annotate the data obtained later or to be obtained in the future with the same standard, without the need to processing and mapping them together again. Thus, transferring cell type annotation from a reference dataset to newly generated query datasets with consistency and reproducibility is increasingly important and necessary. We noted most of the existing AI-based tools although can handle large dataset, they involve information combination and non-linear activation between layers making the final learned features abstract and unable to trace back the input features (including both biological information like genes, and technical information like batch effect, and so on) (as reviewed by refs. ^6–8^ and collected in website https://github.com/OmicsML/awesome-deep-learning-single-cell-papers). For example, the change of dimensions and non-linear aggregation of features throughout the autoencoder’s deep processing stages leads to untraceable and uninterpretable latent space and loss of information and feature resolution^9,10^. In addition, with the increase of non-linear aggregation layers to achieve more powerful learning capability, the model gets deeper meanwhile the contribution from input gets harder to trace, which leads to the loss of interpretability^11^. However, the Transformer framework does not involve dimensionality reduction^10,12^, thus keeping all attention layer traceable to the original input features^13^, thus making the models interpretable. Therefore, we choose Transformer as the framework to develop a new AI-based cell type label transfer tool between a reference dataset and a query dataset, which we named Transformer for One-Stop Interpretably Cell-type Annotation (TOSICA).

TOSICA is a multi-head self-attention network for interpretable cell type annotation in single-cell data and datasets integration simultaneously. By connecting attention to prior biological knowledge and without any batch information, TOSICA interpretably integrates and annotates single-cell data in a batch-insensitive manner while retaining biological variation. Benchmarks and case studies confirm the strength of TOSICA in accuracy and robustness for heterogeneous single-cell data, even in the difficult task of uneven abundance of cell types between reference and query. When tested on many datasets, TOSICA provides the advantage to interpret the attention feature genes and pathways, and surprisingly also automatically filtering out batch effect, potentially as a consequence of direct mapping of cell types to genes (or pathways when using a pathway mask). TOSICA not only met the needs for accurate cell type annotation across different datasets, exceeding existing methods in accuracy, but also often do so with reduced time cost.

## Results

### The structure of TOSICA

TOSICA is an automatic cell-type annotator based on Multi-Head Self-attention^12^. Through supervised training, our model learns the projection function from gene expression to cell type, meanwhile transfers high-dimensional and sparse expression space to low-dimensional and dense feature space.

**Fig. 1:**
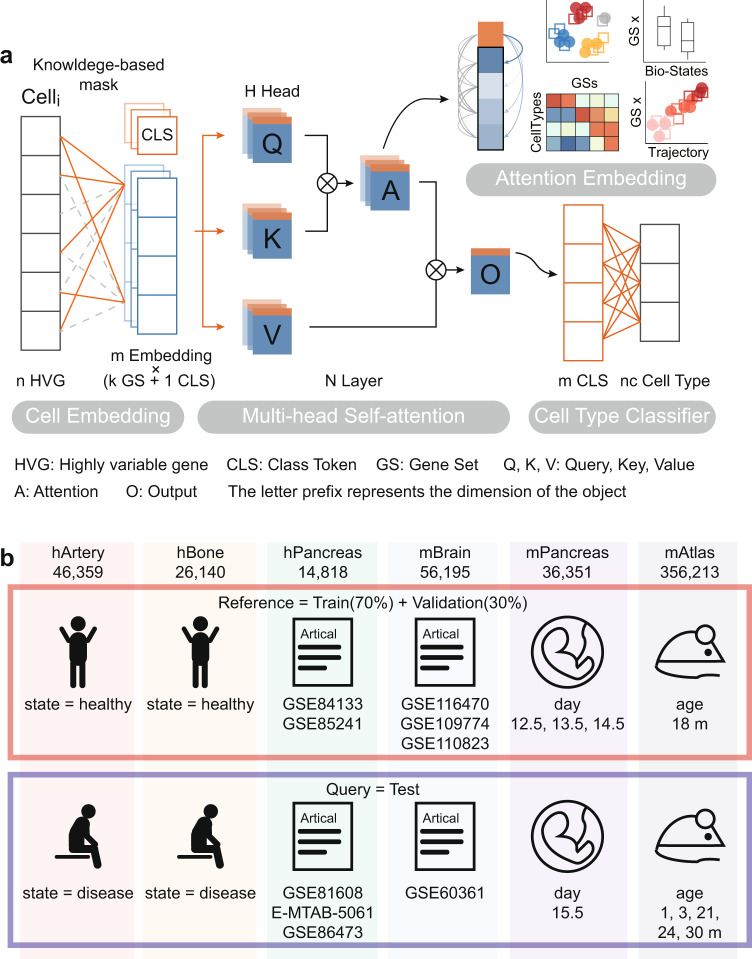
Algorithmic framework of TOSICA. **a** The model is trained on single-cell RNA sequencing data and cell type label for each cell. Based on databases or expert knowledge, masked learnable embeddings are used to convert the reference input data (n genes) to k input tokens representing each gene set (GS), to which class token (CLS) is added. In the attention function, query (Q), key (K), and value (V) matrix are linearly projected from these GSs and CLS combined tokens and the weights (attention, A) is computed by a compatibility function of the Q with the corresponding K, then assigned to each V for computing output (O). In each Multi-head Self-attention layer, the attention function is performed H times in parallel. The CLS of O, considered as latent space of each cell, is used as input of the whole conjunction neural network cell type classifier. Meanwhile, the attention of class (CLS) token to gene set (GS) tokens is referred as attention score and used for cell embedding. **b** hArtery and hBone datasets use healthy samples as training data and predict disease samples. hPancreas and mBrain datasets are split by data source. Training and test data in mPancreas and mAtlas come from different timepoints.

TOSICA is composed of three parts: Cell Embedding layer, Multi-head Self-attention layer, and Cell-Type Classifier (Fig. 1a). The first step of TOSICA is Cell Embedding, which transforms genes into tokens, its transformation matrix is originally a fully connected weight matrix. But transformation matrix is then masked (marked) by a matrix based on expert knowledge (e.g., a gene’s membership to a pathway), only sparse connections among genes and pathways remain in the masked transformation matrix for training and learning (Illustrated in Fig. 1a). Thereby one token only receives information from specific genes and stands for a pathway. This operation is repeated m times in parallel, and all m tokens vectors are merged together. This tokens matrix then is appended with a class token (CLS)^14^, a trainable parameter which then abstracts the information during the following network layers and is used to predict the cell type. Next, this new merged matrix becomes the input of Multi-head Self-attention layer, where the query (Q), key (K), and value (V) matrix are linearly projected from input mentioned before, and each of them can be regarded as a slightly different copy of original input. As biological processes are complex and interactive, there are subtle relationships between pathways, which are calculated by Q and K and referred as attention score (A). It is noteworthy that the attention scores between CLS and pathway tokens mean the importance of the latter to the classification and identification the cell type. Output matrix (O) is the result of operation of A and V, representing a comprehensive score of each pathway and their interacting partners. At this time, CLS in O has collected the information of various pathways, and then transformed to a vector of cell type probabilities. Transformer is successful in interpretability benefited by self-attention mechanism, which calculates the relationship (referred to as “attention”) between tokens of object representation^12^. Just as Vision Transformer calculates attention between an added class token and signatures of pictures to explain which pixels are important for classification^13,14^, TOSICA calculates the attention (relationship mapping) between cell-type classifier token (CLS) and signatures (for example pathway tokens) of cell. In addition, attention scores between CLS and pathway tokens, used as the attention embedding of cells, enable a variety of downstream analyses.

### TOSICA is a universal, accurate and efficient cell type annotator

**Fig. 2:**
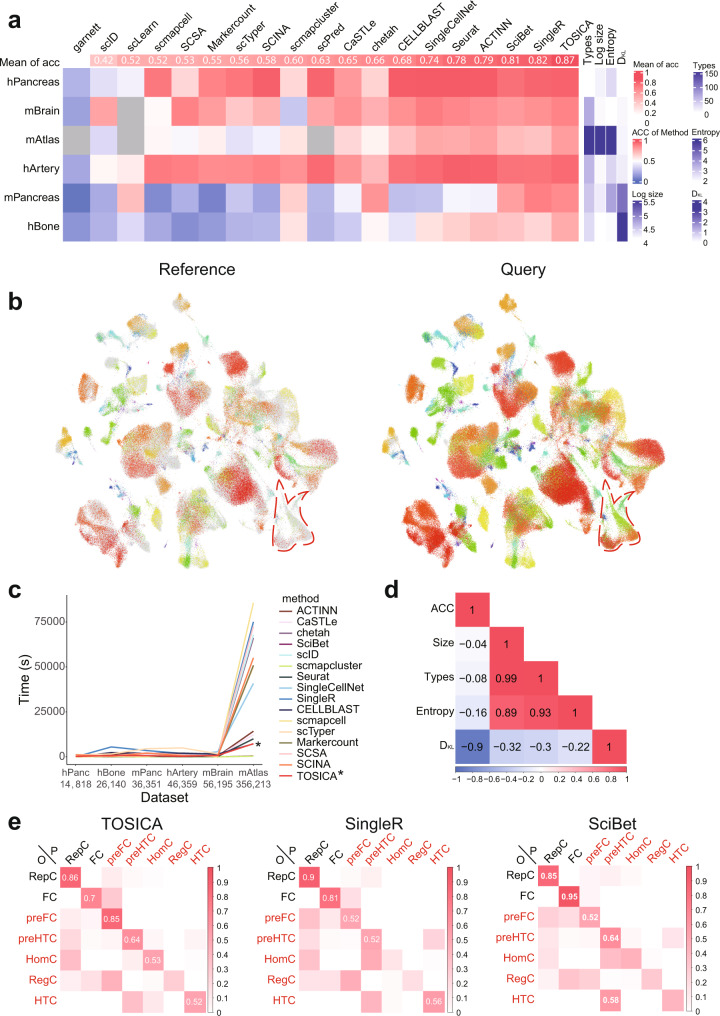
Universality of TOSICA on different datasets. **a** TOSICA ranks first on mean accuracy compared to 18 other cell type annotators on different datasets. Columns are sorted by the mean accuracy of each method on all datasets (top). The number of cell types (Types), number of cells (Log size), Shannon-entropy (Entropy) in reference, and Kullback-Leibler divergence (D_KL_) between reference and query are labeled on the right. Gray means this dataset is too large for this method to deal with. **b** TOSICA succeeds in matching cells in query (mouse age ≠ 18 months) to reference (mouse age = 18 months) on mAtlas as shown by TOSICA attention embedded UMAP. The UMAP is done on the whole mAtlas dataset, including both reference and query. Cells in the reference (left panel) or query (right panel) are colored by cell types while cells in the query (left panel) or reference (right panel) are colored gray. The same types of cells from reference and query are located in the same cluster. Circled cells are rare in reference but clustered correctly in the query by TOSICA. **c** Runtime of TOSICA (marked by *) is relatively stable with increasing data size, and the fourth shortest on mAtlas. hPanc and mPanc stand for hPancreas and mPancreas. **d** D_KL_ has the most negative impact on accuracy. Heatmap shows the correlation between accuracy (ACC) and number of cells (Size), number of cell types (Types), Shannon-entropy (Entropy), and Kullback-Leibler divergence (D_KL_). **e** TOSICA performs better than two other top-ranked methods on five cell types unbalanced between reference and query (red labels). Heatmap shows the proportion of cells in each row with cell type O (original label, shown on the right) is predicted as cell type P (prediction, shown on the top). Cell types are ordered by ratios of their proportions in reference to query. Data are normalized within each row (origin label). Only values >0.5 are labeled. Source data are provided as a Source data file.

We test TOSICA on six different datasets with “ground truth” cell type labels obtained from their original publications: human artery (hArtery)^15^, human bone (hBone)^16^, human pancreas (hPancreas)^17–21^, mouse brain (mBrain)^22–25^, mouse pancreas (mPancreas)^26^, and mouse atlas sequenced by Smart-seq2 and 10X platform (mAtlas)^27^ (Fig. 1b, Supplementary Dataset 1, 2, Supplementary Figs. 1–7), and compare its accuracy with other 18 cell type annotators^2,3,28–43^. The accuracy here is defined as the fraction of cells correctly predicted. The accuracy of TOSICA on every dataset ranks at top 6 (Supplementary Dataset 3), and its mean accuracy of 86.69% is the highest among all 19 methods (Fig. 2a). Although TOSICA ranks fifth and sixth on two easy-to-classify datasets (hArtery and hPancreas), where all top six methods have above 90% accuracy, its accuracy of 93.75% and 95.76% is close to the top-ranked methods (Seurat 96.37% for hArtery and SingleCellNet 97.53% for hPancreas). In contrast, on the datasets that vary significantly on accuracy across methods (hBone, mPancreas, and mAtlas), TOSICA ranks top 2 (Supplementary Dataset 3). Notably, on the biggest dataset mAtlas, which also has the most cell types, TOSICA annotated the cells in query with a high accuracy of 81.06%, while the second best tool ACTINN has an accuracy of 79.57%. And the same types of cells from reference and query are in the same cluster in the TOSICA attention score based UMAP (Fig. 2b). Meanwhile, with the increase of the dataset size, time cost of TOSICA on mAtlas is the fourth shortest and does not explode exponentially like most of the other methods (Fig. 2c).

We then tested the impact of different masks on accuracy (Supplementary Fig. 8a). In order to stimulate the situation of having no expert knowledge, we build two random masks with 1% and 5% reserved connections according to the real-world masks (Supplementary Fig. 8b) to avoid increasing the number of parameters. Random masks usually can result in the similar accuracy as knowledge-based masks, but in the case of mPancreas dataset, the accuracy converges lower with the random mask (Supplementary Fig. 8c). Most importantly, models with random masks need more epochs to converge (Supplementary Fig. 8c). So TOSICA is not limited by expert knowledge and robust to mask choice, and one can choose mask depending on biological context or research interests, but expert knowledge helps to converge to the best model faster.

Since all methods perform relatively badly on hBone dataset, we wonder what characteristics of dataset have the most impact on cell type prediction. We quantify the number of cells (Log size), number of cell types (Types), uneven distribution of cell types (Entropy) in training set, as well as asymmetry of cell types distribution in training and test set (Kullback-Leibler Divergence, D_KL_) (see “Methods”), and calculate their correlation with accuracy. The result shows that, when cell types distribute unevenly between reference set and query set, which is common in real-world, it is difficult for an annotator to predict cell type correctly (PCC between ACC and D_KL_ = −0.9, Fig. 2d). Not surprisingly, the cell type distribution of hBone dataset is the most unbalanced between training and test set (Fig. 2a). On the five cell types, prefibrochondrocytes (preFC), prehypertrophic chondrocytes (preHTC), homeostatic chondrocytes (HomC), regulatory chondrocytes (RegC) and hypertrophic chondrocytes (HTC) that are more unevenly distributed in test or reference set, TOSICA (76.47%) beats the second (SingleR, 63.23%) or third (SciBet, 68.18%) highest mean accuracy methods (Fig. 2e). Altogether, TOSICA has an acceptable time cost on large datasets, while performs better than any other methods on tough tasks, making it a universal cell type annotator.

### TOSICA enables discovery of new cell types

**Fig. 3:**
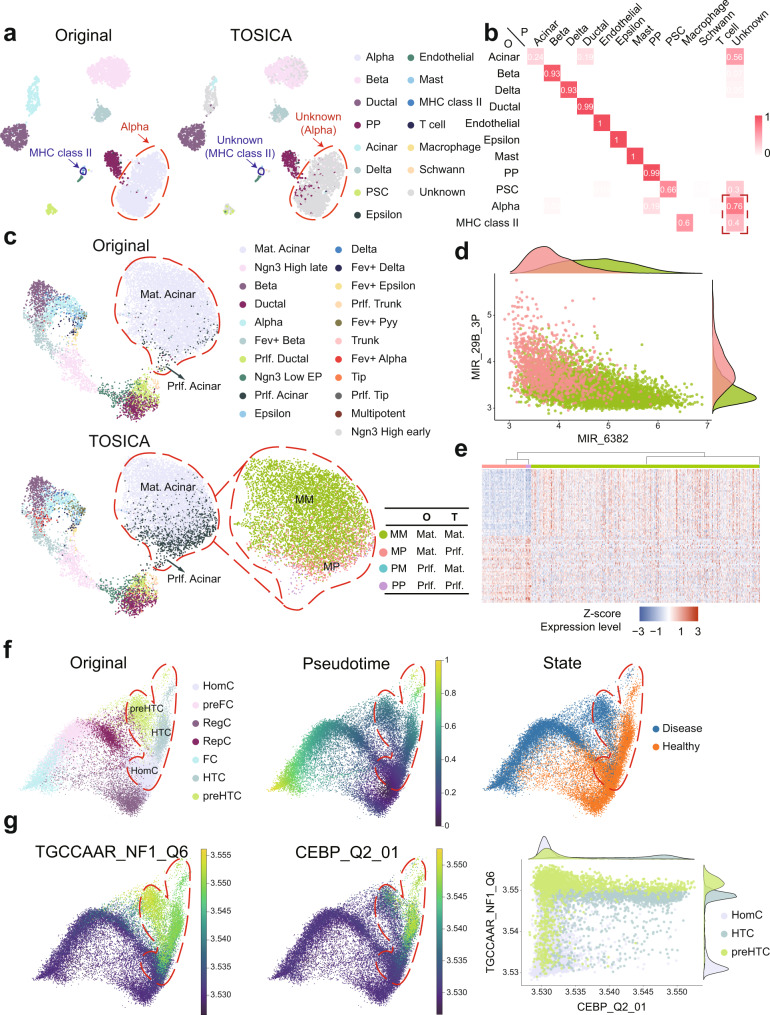
One stop interpretable de novo, high resolution, dynamic, and hierarchical annotation for biological insights by TOSICA. **a** TOSICA successfully isolates and labels the masked alpha cells as ‘Unknown’ cell type. UMAP is based on attention of hPancreas test set. Red circled and marked by red arrows are manually deleted alpha cells and blue circled and marked by blue arrows are MHC class II cells, originally not present in training set. These two kinds of cells are learned as isolated ‘Unknown’ cell types, and are separated by TOSICA attention scores’ UMAP. **b** TOSICA labels most of alpha cell and little other cell types as unknown. Heatmap shows proportion of cells in each row with original label O (original label, shown on the right) predicted as cell type P (prediction, shown on the top). See Supplementary Fig. 9 for comparison to other methods. **c** Some originally labeled mature Acinar (Mat., top) are predicted by TOSICA as proliferative Acinar (Prlf., bottom), red circled. UMAP is based on attention of mPancreas test set. The inset illustrates naming of MM, MP, PM, and PP, originally (O) labeled versus TOSICA (T) labeled. **d** Two pathways’ attention score separate the MM and MP. **e** Hierarchical clustering of DEGs between the originally labeled Mat. Acinar and Prlf. Acinar also groups MM and PM together, and MP and PP together. **f** The proportion changes of 3 cell types in the human bone (red circled) during the transition from healthy to osteoarthritis (OA), shown by diffusion map of hBone, colored by originally labeled cell type (left), pseudotime (middle) and sample status (healthy versus OA (right). Embedding is based on TOSICA attention. **g** High level of NF1 tracks the trajectory from HomC to HTC and preHTC (red circled) shown by diffusion map of hBone, colored by attention score of NF1 pathways (left), and by scatter plot (right), where lower CEBP attention score in preHTC versus HTC associates with OA (middle and right). Source data are provided as a Source data file.

Some cell types are at low abundance in the reference of mAtlas, may be insufficient for training a good predictor, but TOSICA still identifies them well and clusters them together, also separates them from other cell types as much as possible in the query set (Fig. 2b). In a more extreme but common scenario, some cell types have never been seen during training. Thus, we delete the ‘alpha cells’ in reference set of hPancreas to simulate the loss of one high-percentage cell type. As mentioned earlier, the output of TOSICA is the probabilities that a cell is a certain cell type, so when predicting, if the highest probability is below a preset cutoff (0.95), this cell is annotated as ‘Unknown’. As expected, ‘alpha cells’ in the query set of hPancreas are clustered together (Fig. 3a) and 76% of them are labeled as ‘Unknown’ by TOSICA (Fig. 3b), while the rest are labeled as ‘pancreatic polypeptide cell’ (PP), which is also an endocrine cell (Fig. 3b). Three other annotators with high average accuracy, SingleR, SciBet, and ACTINN (Fig. 2a), do not automatically identify ‘alpha’ cells as a new cell type, instead incorrectly label them as ‘PP’, ‘delta’ or ‘beta’ (Supplementary Fig. 9a–c). On contrary, CELLBLAST and chetah, two annotators that actively identify new cell types, label ‘alpha’ cells with 99% and 62% as ‘PP’, with 0 and 37% as a new cell type, respectively (Supplementary Fig. 9d, e). CaSTLe even simply recognizes most of the cells of all cell types as ‘Unknown’, including the cell types that are well-represented in the training sets (Supplementary Fig. 9f). There is also another rare cell type only appeared in query, ‘MHC class II’ cell, and is annotated as ‘macrophage’ or ‘Unknown’ and clustered separately by TOSICA (Fig. 3a, b). Other methods also predict MHC II as ‘macrophage’ or ‘Unknown’ like TOSICA (Supplementary Fig. 9). Since macrophage is one type of MHC II cell, such an annotation is acceptable. Thus, compared to all other methods, TOSICA has a unique ability to accurately discover and annotate new cell types.

### TOSICA provides high resolution and interpretable cell type annotation

Manual annotation of cell types, especially cell subtypes, relies on marker genes selection. However, specificity of marker genes is determined by comparing with the other cell types in the same dataset. Thus, across different datasets, the same cell may have different marker gene sets, thus annotated differently. Here, the annotation standard is variable. A well-trained automatic annotator using uniform biologically relevant standards can avoid the problem of giving the same cell different annotations. In the mPancreas dataset, a class of mature acinar cells (Mat. Acinar) with distribution bias is predicted as proliferative acinar cells (Prlf. Acinar) by TOSICA (Fig. 3c). We examine the reference labeled Mat. Acinar cells that are predicted by TOSICA as Mat. Acinar and Prlf. Acinar, to which we refer as MM (reference Mat. Acinar, TOSICA Mat. Acinar) and MP (reference Mat. Acinar, TOSICA Prlf. Acinar), respectively. Because mPancreas is related to development, we use gene sets representing potential targets of regulation by transcription factors or microRNAs as mask (regulon mask) for TOSICA. We find that MM and MP are distinguished by MIR-6382 and MIR-29B-3P regulons (Fig. 3d), with attention score of MIR-29B-3P higher in MP. Among the genes that are important for MIR-29B-3P regulon based on internal information from TOSICA (Supplementary Fig. 10a), the human homolog of *Sparc* has been reported to increase levels of acinar markers and pro-acinar transcription factors^44^, indicating it is critical role for newborn acinar cells. This also highlights the advantage of hierarchical annotation in not only recovering biological insight at the pathway/regulon level but also at gene level. Principal component analysis of the original expression matrix also shows that MP shares similar PCs with PP (reference Prlf. Acinar, TOSICA Prlf. Acinar) compared with other MM, where the transition ordering is visible on PC1 (Supplementary Fig. 11a). Hierarchical clustering of gene expression matrix further confirms our finding that MP and PP show similar patterns (Fig. 3e). Thus, TOSICA’s gene set attentions automatically distinguish cells originally labeled as Mat. Acinar and Prlf. Acinar, and further identified an intermediate state between the two, which is closer to Prlf. Acinar and incorrectly labeled as Mat. Acinar in the annotation database. This is a manifestation of the high resolution and high accuracy annotation by TOSICA.

### TOSICA enables interpretable dynamic trajectory analysis

Due to the good interpretability of attention score, it can well reconstruct the trajectory and reveal the key pathways in the biological process. Using the top 50 TF regulons attentions to perform the unsupervised pseudotime trajectory analysis, we show the changes of chondrocytes types upon the onset of osteoarthritis (OA) (Fig. 3f). The trajectory (Fig. 3f) is consistent with that obtained by expression matrix^16^. However, different from the routine gene expression-based analysis, TOSICA’s regulon attention-based trajectories directly show that the failure of the transition from NF1 dominance to CEBP regulon dominance characterizes the onset of OA (Fig. 3g), highlighting the biological interpretability and insights generated by TOSICA on the dynamic trajectory. Indeed, the homolog of CEBP has been reported to inhibit proliferation of mouse chondrocytes in vitro^45^, and *Nf1* ablation in *Fgfr1*^*Col2cKO*^ mice reverses their hypertrophic zone phenotype^46^.

### TOSICA is immune to batch effect

Generally, query and reference datasets are generated in different laboratories with different experimental protocols and thus contain batch effects. Batch information is necessary for conventional data-integration method to try to overcome these batch effects, which are difficult to completely remove and mixed up with biological differences. In contrast, despite no batch information is included in either the training set or test set when they both comes from different batches, different studies or subjects (Supplementary Dataset 1), TOSICA can consistently predict cell types with great accuracy (Fig. 2a) and generate batch insensitive embedding, perhaps due to direct mapping of cell types to genes (or pathways when using a pathway mask). We take advantage of an efficient benchmarking tool scIB^47^ to assess TOSICA and other integration methods on 5 datasets via batch average silhouette width (batch ASW), which measures the relationship between the within-cluster distances of a cell and the between-cluster distances of a cell to the closest cluster to evaluate batch effect removal, and global cluster matching (normalized mutual information, NMI), which compares the overlap of two clusters to evaluate biological conservation. Larger values of batch ASW and NMI represent stronger ability of batch effect removal and biological conservation, respectively^47^. On 2 of the 5 test datasets, which have more cells, the batch ASW of TOSICA ranks in the top 2 and is only slightly lower (0.02–0.06 or 2.1–5.6%) than the top 1 method’s batch ASW (Supplementary Fig. 11b). Meanwhile, biological NMI of TOSICA ranks within top 5 among 14 methods on each dataset. Conspicuously, while scGen and Seurat show excellent ability on datasets with fewer batches and cells, neither of them works on mouse atlas dataset, on which TOSICA ranks at the top in both batch effect removal and biological conservation (Supplementary Fig. 11b). Also, TOSICA is robust against the choice of masks in its of batch effect removal ability, except batch effects removal ability is unexpectedly slightly stronger when using random masks, and it is expected that some knowledge-based masks are better than others for a specific dataset, for example for hBone (Supplementary Fig. 11b). These results indicate that TOSICA is insensitive to batch effect and good at biological conservation, and excels on large datasets with many batches, especially considering that we never provide batch information to it.

### Interpretability of TOSICA is hierarchical

All previous cell type annotators are gene-based, thus reveal little on the biological insight behind the cell type marker genes, many more subsequent analyses are needed to infer the potential enriched pathways and regulators behind the marker genes. Instead, by embedding genes to higher level of biological processes tokens, TOSICA directly learns the biological processes and signaling pathways giving rise to the cell types, thus separating cell types, including new cell types (Fig. 2d) with accurate high-resolution annotation (Fig. 3d) and allowing direct trajectory regulation discovery (Fig. 3g), while immune to batch effect (Supplementary Fig. 11b). This high-level attention framework not only allows interpretability but is essential for the high accuracy of TOSICA (Fig. 2a). Furthermore, as shown by the discovery of MIR-29B-3P regulon (Fig. 3d) and its important target gene *Sparc* in the development of acinar cell (Supplementary Fig. 10a), the interpretability does not stop at the high-level structures, the important low-level entities, genes, that significantly contribute to these high-level annotations are also available from the networks within TOSICA (Supplementary Fig. 10), and can be revealed simultaneously thus generating a comprehensive hierarchical annotation structure.

### TOSICA parses tumor infiltrating myeloid cells heterogeneity with high resolution

**Fig. 4:**
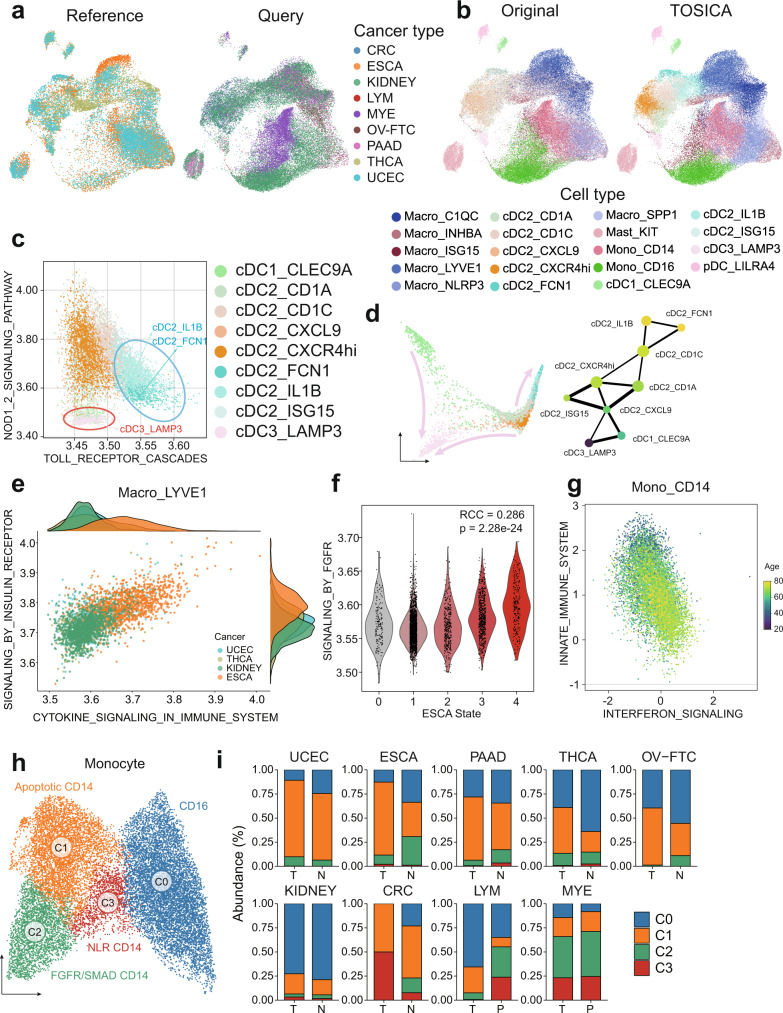
TOSICA resolves pan-cancer tumor infiltrating myeloid cell heterogeneity. **a**, **b** TOSICA predicts cell types reliably across different cell types even when the reference and query contain no overlapping cancer types as shown by TOSICA attention embedded UMAP. UMAP is colored by the cancer types in the reference (3, left panel in **a**), in query (6, right panel in **a**), and by cell types in the query as originally labeled (left panel in **b**) and predicted by TOSICA (right panel in **b**). **c** cDC2_FCN1, cDC2_IL1B, and cDC3_LAMP3 distinguish from other cell types in attention scores of 2 REACTOME pathways. Each dot represents one cell and is colored by cell types. **d** Three developmental trajectories from cDC2_CXCL9 and cDC1_CLEC9A to cDC3_LAMP3 and cDC2 to cDC2_FCN1, cDC2_IL1B delineated by TOSICA attention embedded diffusion map (left) and partition-based graph abstraction (PAGA) (right). Edge weights in PAGA represent confidence for the connections between cell types, colored by pseudotime. **e** Macro_LYVE1 of ESCA distinguish from that of other cancers in attention scores of 2 REACTOME pathways. **f** Attention score of SIGNALING_BY_FGFR increases with advanced stage of ESCA. Statistical test is two-sided. **g** INNATE_IMMUNE_SYSTEM is downregulated and INTERFERON_SIGNALING is upregulated during aging in Mono_CD14. Dots are colored by age. **h** Attention score based UMAP identifies 4 subtypes of monocytes. **i** The distribution of the 4 monocyte subtypes changes with tumor (T) versus matching normal (N) tissues or peripheral blood (P) in different cancer types. Source data are provided as a Source data file.

One of the most common demands in single-cell analysis is the transfer of identified cell population from an original reference to newly generated data, which may come from different batch and biological state (e.g., disease). To demonstrate the applicability and interpretability of TOSICA on such a task, we prepare two sets of pan-cancer tumor infiltrating immune cells data, myeloid^48^ and T^49^ cells, respectively. In the myeloid dataset, a total of 71,159 myeloid cells come from tumors, adjacent non-cancer tissues, peripheral blood of 43 patients across 9 common cancer types. Among them, kidney cancer (KIDNEY, 28,930 cells), uterine corpus endometrial carcinoma (UCEC, 9816 cells) and esophageal carcinoma (ESCA, 8154 cells) are used as reference dataset (Fig. 4a) and myeloma (MYE, 7861 cells), thyroid carcinoma (THCA, 5939), ovarian or fallopian tube carcinoma (OV-FTC, 4002 cells), pancreatic adenocarcinoma (PAAD, 3093 cells), colon cancer (CRC, 2725 cells), and lymphoma (LYM, 639 cells) are used as query dataset (Fig. 4a, b). REACTOME pathway^50^ knowledgebase is used to build the model. Then, 8 evaluation metrics (ASW, graph connectivity and k-nearest-neighbor batch effect test (kBET) for batch effect removal and NMI, Adjusted Rand Index (ARI), ASW and isolated label F1 score for biological variation retention) are computed to verify the integration ability by scIB^47^. scIB ranks TOSICA the second out of all 11 applicable data integration methods evaluated on all metrics combined (overall score = 0.6 × biology conservation + 0.4 × batch removal) (Supplementary Fig. 12a). Note that Seurat-based methods, including Seurat v3 CCA and Seurat v3 RPCA, are unable to integrate datasets from more than 85 batches, these methods are thus not applicable for comparison.

On the cDCs populations, TOSICA reveals that the same cDC subsets from different tumor types are clustered together (Supplementary Fig. 12b), which is consistent with previous observations^48^. In particular, TOSICA detects a pair of population-specific pathways (NOD1/2 SIGNALING PATHWAY and TOLL RECEPTOR CASCADES) that separate inflammation-related cDCs (cDC2_FCN1 and cDC2_IL1B) and a mature cDC subset (cDC3_LAMP3), which broadly present in tumor microenvironment (TME) from the rest of cDCs (Fig. 4c). This is in agreement with previous observations showing low expression of Toll-like receptor (TLR) signaling genes and low innate immune activity of cDC3_LAMP3^48^ and the “pro-inflammatory” properties of cDC2_FCN1 and cDC2_IL1B in blood^51^. As interpretable trajectories, the diffusion map^52^ based on TOSICA attention embedding confirms two potential origins of the cDC3_LAMP3 from cDC1s and cDC2-CXCL9, as previously suggested^48^ (Fig. 4d). Furthermore, the map reveals another state transition path from cDC2 to pro-inflammatory cDC2 subtypes (Fig. 4d), which has not been observed in the previous analysis^48^. Such an observation is further supported by partition-based graph abstraction (PAGA) analysis and diffusion pseudotime reconstruction, when cDC3_LAMP3 is regarded as the root of the lineages (Fig. 4d).

On the LYVE1+ resident tissue macrophages (RTMs), which functions to restrain inflammation and fibrosis in multiple human tissues^53^, TOSICA shows significant heterogeneity of attention scores in Macro_LYVE1 across different caner types. TOSICA attention scores reveal that ESCA separates from other cancers in cytokine signaling and insulin signaling pathway (Fig. 4e), hinting at higher inflammatory state of LYVE1+ RTMs in ESCA, which was not observed in the original study.

Next, we examine whether TOSICA is able to detect the state shift during disease progression and aging within the same cell type. TOSICA attention scores show a significant upregulated of FGFR signaling pathway (Fig. 4f, RCC = 0.29 *p* = 2.28e−24) and downregulated of interferon signaling with advanced stage of ESCA in LYVE1+ RTMs (Supplementary Fig. 12c, RCC = −0.30, *p* = 1.38e−27). Besides, the loss-function of innate immune system with aging^54^ is detected in CD14+ Mono (RCC = −0.26, *p* = 2.68e−177), which is accompanied by slight up-regulation (RCC = 0.14, *p* = 2.0e−47) of IFN signaling (Fig. 4g). Such pathway level association with disease progression or aging have not been observed in the previous analysis with other methods. The 5 important genes for these two regulon tokens in TOSICA include the well-known inflammatory genes *NLRP3* and *IFITM3* (Supplementary Fig. 12d).

Furthermore, benefiting from its high resolution, TOSICA identifies several subtypes of monocytes that have not been discovered in the original publication^48^ (Fig. 4h), all having their own biological signatures and potentially different functions (Supplementary Fig. 12e). Subtype C1 apoptotic CD14 is generally enriched in tumor tissues (paired t-test *p*-value = 0.021), especially in ESCA (paired t-test *p*-value = 0.0012) when compared to the matching normal tissues, while C0 CD16 mainly resides in non-tumor tissues of the same type (paired t-test *p*-value = 0.0015) (Fig. 4i).

Overall, TOSICA accurately annotates query tumor infiltrating myeloid cell types. With high biological resolution and batch insensitivity of attention (Supplementary Fig. 12a), TOSICA reveals many novel dynamic and functional status of single cells, with their key contributors hierarchically annotated at both pathway and gene levels to guide further experimental explorations.

### TOSICA reveals tumor infiltrating T cells dynamics

Discovering the origin of tumor infiltrating T cells is important to cancer immune therapy. Here on a tumor infiltrating T cells dataset, a total of 109,389 CD8+ T cells and 79,303 CD4+ T cells derived from the tumors, adjacent non-cancer tissues, peripheral blood of 48 patients across 11 common cancer types, in which THCA (56,958 cells), UCEC (32,655 cells) and breast cancer (BC, 7354 cells) are used as reference dataset (Supplementary Fig. 13a, b) and renal cancer (RC, 26,649 cells), ESCA (24,884 cells), multiple myeloma (MM, 12,274 cells), B-cell lymphoma (BCL, 11,956 cells), pancreatic cancer (PACA, 9860 cells), ovarian cancer (OV, 4523 cells), fallopian tube carcinoma (FTC, 1037 cells) and cholangiocarcinorma (CHOL, 542 cells) are used as query dataset (Supplementary Fig. 13a, b). REACTOME pathway knowledgebase is used as mask in TOSICA. On this dataset, TOSICA ranks second out of all 10 applicable methods on the combined effectiveness in batch effect removal and biological variation retention (Supplementary Fig. 13c). In addition, the runtime of TOSICA is the shortest (minutes), while it takes scGen nearly five days to finish (Supplementary Fig. 13c).

Diffusion map based on TOSICA attention embedding recapitulates the previous observation^49^ that CD4+ T cells develop from naïve T cells to Temra cells, TFH/TH1 cells, or TNFRSF9+ Treg cells, separately (Supplementary Fig. 14a–c). Along this transition process, many interleukin signaling pathway and cytotoxic effector molecules (Supplementary Fig. 14c)—including IL2, IL1, IL6, TLR, NETRIN1, CTLA4, and CBL related pathway—significantly increase (FDR < 0.001, generalized additive model) and MHCI/II, IL7 and TGFb pathways decrease (FDR < 0.001, generalized additive model). In CHOL, UCEC, PACA, and ESCA, the tumor infiltrating CD4+ T cells are more likely to develop along Treg path rather than Temra path (Supplementary Fig. 14d). Likewise, attention score based UMAP shows that GXMK+ Tex cells, not terminal Tex cells as previously assumed^49^, are the common end point of the two state transition path from naïve CD8+ T cells: the first path going through GZMK+ Tem cells, and the second going through ZNF683+ Trm and terminal Tex cells, which are previously considered to be the end of the transition process of the two dynamic path^49^ (Supplementary Fig. 14e, f). Besides, TOSICA also reveals specific inflammatory and metabolic pathways enriched for each cell type in the transition process (Supplementary Fig. 14g).

In this case, TOSICA demonstrates its advantage compared to other cell type annotators in uncovering previously unknown dynamic trajectories of cells.

### TOSICA hierarchically interprets the immune response of patients with COVID-19 and SLE

**Fig. 5:**
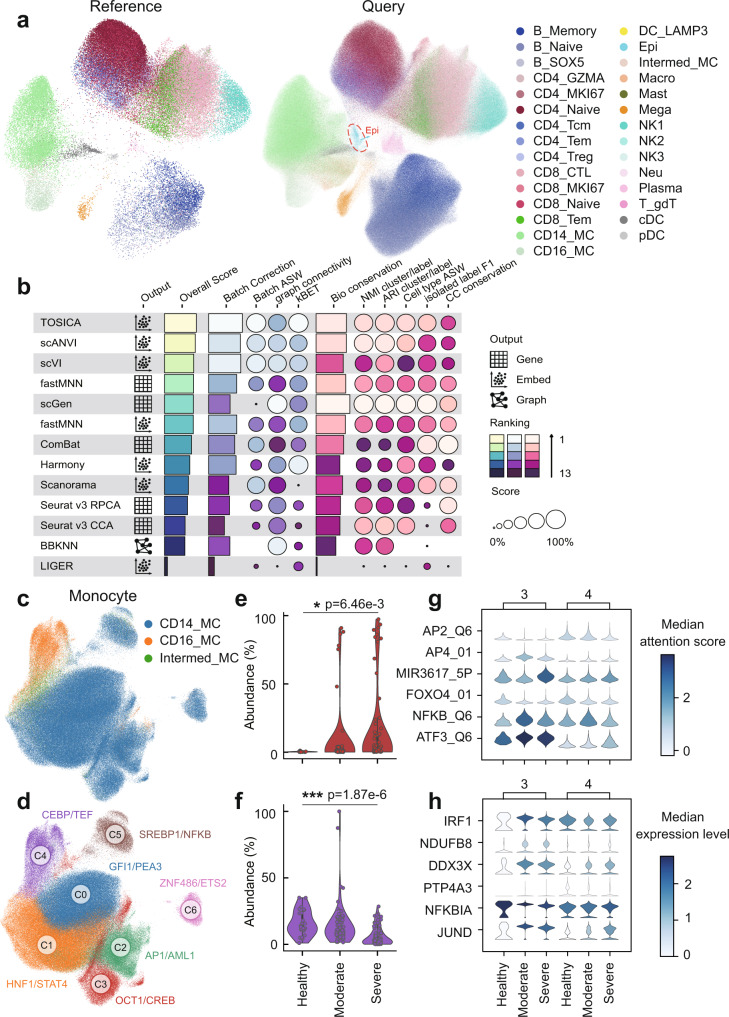
TOSICA reveals change in transcription factor activity during moderate and severe COVID-19. **a** TOSICA predicts cell types reliably across different cell types even when using healthy individuals as reference (left) and COVID19 patients as query (right). Colors denote 29 origin labels. Red circled cell types are unique in query. **b** Comparison of integration accuracy on query data places TOSICA first among 13 methods. Each score is minimum–maximum scaled between 0 and 1. Overall scores are computed using a 40:60-weighted mean of batch correction and bio-conservation scores. **c**, **d** TOSICA attention score based UMAP predicts 3 known (**c**) and 6 novel (**d**) monocyte types. **e**, **f** Subtype 3 monocytes increases (**e**) and subtype 4 decreases (**f**) in abundance from healthy (*N* = 25), to moderate (*N* = 79), and to severe (*N* = 91) COVID-19. Statistical test is two-sided. * RCC *p* < 0.05; ***p < 0.001. **g** TOSICA attention score of 6 transcription factors distinguishes subtype 3 and 4 monocytes across different states of COVID19. **h** The expression levels of major targets of the 6 TFs (**g**) generally show consistent trends with TFs attention score. Source data are provided as a Source data file.

To demonstrate large-scale interpretable biomedical application of TOSICA, we use it to determine the transcriptional programs of the cellular response to COVID-19 infection. We reanalyze a large-scale COVID-19 single cell transcriptome atlas of PBMC^55^, in which parts of healthy control from Wuhan, Beijing, Harbin and Suihua cohorts (52,836 cells) are used to train the TF regulon masked TOSICA and the rest of healthy control and COVID-19 positive patients from 10 city cohorts (1,409,866 cells) are used as query dataset (Fig. 5a). Among all cell types, DC_LAMP3, Epi and Mast are unknown cell types for reference but TOSICA can still identify them de novo as an isolated cluster on UMAP (Fig. 5a and Supplementary Fig. 15a) with little batch effect (Supplementary Fig. 15b). Furthermore, 8 evaluation metrics (3 for batch effect removal and 5 for biological variation retention) are computed to verify the integration ability by scIB^47^, which ranks TOSICA the first out of all 13 applicable methods evaluated on combined effectiveness in batch effect removal and biological variation retention (Fig. 5b). We then evaluate the significantly enriched TFs within NK cells (Supplementary Fig. 15c), CD8+ T cells, CD4+ T cells, B cells and myeloid cells (Supplementary Fig. 15d). Compared with the expression of marker genes, TFs attention score of MYOD_01 can separately label NK cells (Supplementary Fig. 15c), while the expression of the known NK cell marker gene *NKG7*, mixes NK cells with CD8+ T cells (Supplementary Fig. 15e).

On monocytes (Fig. 5c), the major inflammatory cell types, TOSICA identifies 7 subtypes of monocytes, one for CD16+ monocytes and 6 for CD14+ monocytes (Fig. 5d). Among them, C3 population (high activity of OCT1 and CREB) decreases and C4 population (high activity of CEBP and TEF) increases during COVID-19 progression from healthy to moderate to severe (Fig. 5e, f). TOSICA’s TF regulon attentions in C3 and C4 show that AP2_Q6 and FOXO4_01 have low activities and AP4_01, MIR3617_5P, NFKB_Q6, and ATF3_Q6 have upregulated activities during COVID-19 disease progression (Fig. 5g). Their typical target genes indeed show a similar expression pattern (Fig. 5h).

As a final case, we use TOSICA to assist with interpretable cell type annotation from established independent reference model and analyze cell response heterogeneity. As example, we use reference model trained in the above COVID-19 analysis to map a query PBMC dataset of eight patients with systemic lupus erythematosus (SLE) whose cells were either untreated (control) or treated with interferon (IFN-β)^56^ (Supplementary Fig. 16a). Not surprisingly, our model is able to identify the cell state transition under IFN-β treatment on monocytes (Supplementary Fig. 16b). Differential TF attention can distinguish different cell types (Supplementary Fig. 16c). Between IFN-β and control conditions in all cells, the top 25 differentially active TFs, including the top-ranked SREBP (Supplementary Fig. 16d), are consistent with previously reported interferon induction of lipogenesis^57^, which has not been described in the previous scRNA-seq analysis^56^. Consistent with this finding, in each cell types, the activity of SREBP and SREBP1 are also upregulated and FOXO1/3 are downregulated by IFN-β, especially in myeloid cells (Supplementary Fig. 16e). Furthermore, several pairs of population-specific TF activities can separate IFN-β-related CD14+ Mono and B cells from untreated cells (Supplementary Fig. 16f, g).

In this example, TOSICA preserves cell type response to disease and drug interference after reference mapping. The intelligible and interpretable high-resolution annotation transfer between completely independent studies on different biological processes is demonstrated, thus allowing interdisciplinary data integration of single-cell studies.

## Discussion

In this study, we develop and establish TOSICA, a Transformer-based cell type annotation and integration tool that offers accurate, transferrable, high-resolution, batch insensitive, biologically interpretable cell type annotations under many scenarios, including but not limited to new cell type discovery, dynamic trajectory analysis, cross platform, and population dataset integration. The high accuracy and batch-insensitivity of TOSICA can be mainly attributed to the attention layers and tokens masked by high-level biologically relevant pathways or regulons in the Transformer architecture, which allow TOSICA to focus on biologically relevant interacting genes, pathways or regulons, instead of individual genes that are susceptible to random noise and/or batch effects. By doing so, new cell types, high-resolution subtypes, and their dynamic behaviors are also recognized by their biologically relevant and interacting signatures rather than random noise and/or batch effects, meanwhile the annotations are, intrinsically by default, biologically relevant and interacting signatures generated by the attention layer. The various systems level comparisons with existing methods and case-by-case close examination of different datasets and tasks demonstrate the accuracy, robustness, flexibility, and generalizability of TOSICA as an indispensable new tool for advancing the single-cell studies. As an innovative application of Transformer architecture in single-cell omics data analysis, TOSICA creates an unprecedented opportunity toward effectively and interpretably annotating cell types across large-scale datasets in one step. The whole package of TOSICA, along with tutorials and demo cases, is available online at https://github.com/JackieHanLab/TOSICA^58^ for the community. We also provide a simple workflow schematic of how to use the TOSICA toolkit (Supplementary Fig. 17).

## Methods

### TOSICA model

For each cell, expression levels of *n* genes (${{{{{{\bf{e}}}}}}\in {\mathbb{R}}}^{n}$) are first embedded into *k* tokens (${{{{{{\bf{t}}}}}}\in {\mathbb{R}}}^{k}$) using linear transformation weight (**W**), which will be learned during training.

To achieve that every token represents a different pathway, the weight matrix of linear transformation is masked, only if these genes belong to the pathway, the connection can be saved. Thus, we generate a mask matrix (**M**) using expert knowledge, **M** is composed of 0 or 1 and has the same dimension as **W**. The masked linear transformation weight (**W**′) is the product of the corresponding positions of **W** and **M**.

1 $${{{{{{\bf{W}}}}}}}^{\prime}={{{{{\bf{W}}}}}}*{{{{{\bf{M}}}}}}$$

2 $${{{{{\bf{t}}}}}}={{{{{{\bf{W}}}}}}}^{\prime} \cdot {{{{{\boldsymbol{e}}}}}}$$

Then the embedding operation is repeated *m* times in parallel to increase the dimensions of embedding space, where *m* is a hyper-parameter that can be manually set, with a default of 48. Then all **t**s are concatenated by column.

3 $${{{{{\bf{T}}}}}}{{=}}{{{{{\rm{columnbind}}}}}}\left({{{{{{\bf{t}}}}}}}_{1}{{,}}\,{{{{{{\bf{t}}}}}}}_{2}{,}\ldots {,}\,{{{{{{\bf{t}}}}}}}_{m}\right){,}\,{{{{{\bf{T}}}}}}{\in {\mathbb{R}}}^{k\times m}$$

Here, **T** (${{{{{{\bf{T}}}}}}\in {\mathbb{R}}}^{k\times m}$) represents the pathway token matrix. Each row in **T**, the so-called token, stands for a pathway.

Following, a learnable parameter class token (**CLS**) is concatenated to **T** at the top by row, and generates the input matrix (**I**).

4 $${{{{{\bf{I}}}}}}{{{{{\boldsymbol{=}}}}}}{{{{{\rm{rowbind}}}}}}\left({{{{{\bf{CLS}}}}}}{{{{{\boldsymbol{,}}}}}}\,{{{{{\bf{T}}}}}}\right){{{{{\boldsymbol{,}}}}}}\,{{{{{\bf{CLS}}}}}}\,{\in {\mathbb{R}}}^{m},\,{{{{{\bf{I}}}}}}\,{\in {\mathbb{R}}}^{1+k\times m}$$

An attention function can be described as mapping a query and a set of key-value pairs to an output^12^. In Multi-head self-attention layer, the query (**Q**), key (**K**), and value (**V**) matrix are separately linearly projected from input matrix (**I**) mentioned above, and the linear projection weights are referred as **W**_*q,k,v*_.

5 $$\begin{array}{c}{{{{{\bf{Q}}}}}},\,{{{{{\bf{K}}}}}},\,{{{{{\bf{V}}}}}}={{{{{{\bf{W}}}}}}}_{q,k,v}\cdot {{{{{\bf{I}}}}}}\\ {{{{{\bf{Q}}}}}}{{{{{\boldsymbol{,}}}}}}\,{{{{{\bf{K}}}}}}{{{{{\boldsymbol{,}}}}}}\,{{{{{\bf{V}}}}}}{{\mathbb{\in }}{\mathbb{R}}}^{1+k\times m}\end{array}$$

Then attention (**A**) matrix is computed by **Q** with the corresponding **K**, scaled by the inverse of the square of dimension of **K** (*d*_*k*_) and activated by softmax function.

6 $${{{{{\bf{A}}}}}}={{{{{\rm{softmax}}}}}}\left(\frac{{{{{{\boldsymbol{Q}}}}}}\cdot {{{{{{\bf{K}}}}}}}^{{{{{{\rm{T}}}}}}}}{\sqrt{{d}_{k}}}\right)$$

where, *d*_*k*_ = *m*, and

7 $${{{{{\rm{softmax}}}}}}\left({{{{{{\bf{z}}}}}}}_{i}\right)=\frac{{{\exp }}\left({{{{{{\bf{z}}}}}}}_{i}\right)}{{\sum }_{j}{{{{{{\bf{z}}}}}}}_{j}}$$

Then **A** is assigned to each **V** for calculate output (**O**).

8 $${{{{{\bf{O}}}}}}={{{{{\rm{Attention}}}}}}\left({{{{{\bf{Q}}}}}},\,{{{{{\bf{K}}}}}},\,{{{{{\bf{V}}}}}}\right)={{{{{\bf{A}}}}}}\cdot {{{{{\bf{V}}}}}}$$

It is reported that instead of performing a single attention function, it beneficial to linearly project the queries, keys and values *H* times, which is the so called muti-head and each repeat is a head, with different, learnable linear projections to *d*_*q*_, *d*_*k*_, and *d*_*v*_ dimensions by **W**^*Q,K,V*^, respectively^12^.

9 $${{{{{\bf{O}}}}}}={{{{{\rm{MultiHead}}}}}}\left({{{{{\bf{Q}}}}}},\,{{{{{\bf{K}}}}}},\,{{{{{\bf{V}}}}}}\right)={{{{{{\bf{W}}}}}}}^{O}\cdot {{{{{\rm{columnbind}}}}}}\left({{{{{{\bf{head}}}}}}}_{1},\ldots,\,{{{{{{\bf{head}}}}}}}_{H}\right),\,{{{{{\bf{O}}}}}}\,{\in {\mathbb{R}}}^{1+k\times m}$$

10 $${{{{{{\rm{where}}}}}},\,{{{{{\bf{head}}}}}}}_{i}={{{{{\rm{Attention}}}}}}\left({{{{{{\bf{W}}}}}}}_{i}^{Q}\cdot {{{{{\bf{Q}}}}}},\,{{{{{{\bf{W}}}}}}}_{i}^{K}\cdot {{{{{\bf{K}}}}}},\,{{{{{{\bf{W}}}}}}}_{i}^{V}\cdot {{{{{\bf{V}}}}}}\right)$$

The **CLS** of **O** is used as input of a fully connected network and followed by a softmax function to obtain the probability of cell types (${{{{{{\bf{p}}}}}}\in {\mathbb{R}}}^{{nc}}$, *nc* = number of cell types).

11 $${{{{{\bf{p}}}}}}={{{{{\rm{softmax}}}}}}\left({{{{{{\bf{W}}}}}}}_{p}\cdot {{{{{\bf{CLS}}}}}}\right)$$

In addition, attention weights (or named as attention score) of **CLS** to pathways are abstract as low-dimensional feature of cell^13^.

In order to prevent overfitting, we refer to a previous research^12^, and introduce residual connection. In order to increase the model’s ability to learn complex information, we add two more full-connected layers after the attention sub layer (Supplementary Fig. 18).

### Knowledge-based mask matrix

The mask matrix used in this work is based on knowledge datasets from GSEA (http://www.gsea-msigdb.org/gsea/downloads.jsp). In particular, we map the input genes to selected gene sets (gmt files), such as c2.cp.reactome.v7.5.1.symbols.gmt and c3.all.v7.5.1.symbols.gmt. Two parameters are optional: a maximum number of genes in each gene set (default as 300) and a maximum number of gene sets (default as 300). The mask matrix is in the form of a binary matrix **M** with columns corresponding to numbers of gene sets and rows corresponding to genes, with **M**_*i,j*_ = 1 if the gene *i* belongs to the gene set *j*, otherwise **M**_*i,j*_ = 0. Then, the matrix is sacked *m* times (dimension of embeddings) to generate gene set tokens from gene input, where *m* where can be customized with a default of 48.

### Model training

We choose different studies or biological states to split the training and test set (Supplementary Dataset 1), and 30% of training set is divided as validation set.

The accuracy is determined as the ratio of samples predicted correctly over all samples. The loss is calculated by cross entropy loss function. Stochastic gradient descent (SGD) is chosen as optimizer, and we use cosine learning rate decay to avoid too large steps in late stage of training. Typically, TOSCIA converges within 20 epochs.

### Other annotation methods

For all methods used for comparison, we provided them the same training (reference) dataset and test (query) dataset. And they are run using their recommended default parameters. The majority of the methods have built-in normalization. So, we provided each method with the raw count data or log_10_(1e4*count +1) according to their description.

### Quantify the characteristics of datasets

‘*Log size*’ is computed as below:

12 $${Log\; size}={{{{{{\rm{log }}}}}}}_{10}\left({number}\,{of}\,{samples}\,{in}\,{dataset}\right)$$

‘*Types*’ equals the number of cell types.

13 $${Types}={number}\,{of}\,{cell}\,{types}$$

‘*Entropy*’ is defined as bellow:

14 $${Entropy}=\mathop{\sum }\limits_{i=1}^{{nc}}{{{{{{\rm{log }}}}}}}_{2}\left({p}_{i}\right)\cdot {p}_{i}$$

15 $${{{{{\rm{where}}}}}},\,{p}_{i}=\frac{{{{{{{\rm{number}}}}}}}\,{{{{{{\rm{of}}}}}}}\,{{{{{{\rm{samples}}}}}}}\,{{{{{{\rm{labelled}}}}}}}\,{{{{{{\rm{as}}}}}}}\,{{{{{{\rm{cell}}}}}}}\,{{{{{{\rm{type}}}}}}}\,i\,{{{{{{\rm{in}}}}}}}\,{{{{{{\rm{training}}}}}}}\,{{{{{{\rm{set}}}}}}}}{{{{{{{\rm{number}}}}}}}\,{{{{{{\rm{of}}}}}}}\,{{{{{{\rm{all}}}}}}}\,{{{{{{\rm{samples}}}}}}}\,{{{{{{\rm{in}}}}}}}\,{{{{{{\rm{training}}}}}}}\,{{{{{{\rm{set}}}}}}}}$$

We use Kullback-Leibler Divergence (*D*_*KL*_) to evaluate the unbalance between reference and query sets:

16 $${D}_{{KL}}=\mathop{\sum }\limits_{i=1}^{{nc}}{{{{{{\rm{log }}}}}}}_{2}\left({q}_{i}\right)\cdot {p}_{i}-\mathop{\sum }\limits_{i=1}^{{nc}}{{{{{{\rm{log }}}}}}}_{2}\left({p}_{i}\right)\cdot {p}_{i}$$

where, *p*_*i*_ is same as (15) and

17 $${q}_{i}=\frac{{{{{{{\rm{number}}}}}}}\,{{{{{{\rm{of}}}}}}}\,{{{{{{\rm{samples}}}}}}}\,{{{{{{\rm{labelled}}}}}}}\,{{{{{{\rm{as}}}}}}}\,{{{{{{\rm{cell}}}}}}}\,{{{{{{\rm{type}}}}}}}\,i\,{{{{{{\rm{in}}}}}}}\,{{{{{{\rm{test}}}}}}}\,{{{{{{\rm{set}}}}}}}}{{{{{{{\rm{number}}}}}}}\,{{{{{{\rm{of}}}}}}}\,{{{{{{\rm{all}}}}}}}\,{{{{{{\rm{samples}}}}}}}\,{{{{{{\rm{in}}}}}}}\,{{{{{{\rm{test}}}}}}}\,{{{{{{\rm{set}}}}}}}}$$

### Data analysis

Python version 3.8.11 and R version 4.0.5 were used for downstream analysis with the following packages: torch (version 1.7.1), scanpy (version 1.7.1), Seurat (version 4.1.0), ggplot2 (3.3.5), ComplexHeatmap (2.10.0), gam (1.22), and their dependent packages.

### Attention embedding preprocessing

The preprocessing of attention matrix is similar to that of the scanpy^59^ pipeline for scRNA-seq data. First, the matrix is normalized by library-size correction using default size factor 10,000. Then, all attentions are identified as input to perform PCA analysis. And then PCA matrix is used to build nearest neighbor graph, which is further embedded in two-dimensional UMAP for visualization.

### Benchmarking data integration

scIB^47^ is used to benchmark data integration ability (version 1.0.0). For existing methods, default parameters are used and only ‘full features’ and ‘unscaled’ model are used for comparing. For TOSICA, the raw attention embedding is used as input to scIB.

The study information in human pancreas and mouse brain dataset, donor information in human artery, human bone, mouse atlas, cancer and COVID-19 dataset are used for batch effect removal assessment. The cell type information in all datasets is used for biological conservation evaluation.

### Identification of signature attentions of cell types and sub-clusters

The signature attentions of cell types are identified based on Wilcoxon rank-sum (Mann–Whitney-U) test. Same as scanpy, attention scores are normalized to 1e4 and logarithmized. Then, sc.tl.rank_genes_groups(method=‘wilcoxon’) is used for finding marker attentions. *P*-values are adjusted by the Benjamini–Hochberg (BH) method.

As for sub-cluster identification, the cells of interest are selected, normalized, and logarithmized alone. All attentions are identified as input to perform PCA analysis. And then PCA matrix is used to build nearest neighbor graph, which is then used to find clusters by Louvain algorithm with parameter “resolution” = 0.3 to identify sub-clusters.

### Genes’ importance to a pathway token

The importance of genes to pathway tokes are computed from the linear transformation layer. Each gene’s weight for a token is calculated as the mean of the absolute value of weights in all embedding dimensions.

### Cell differentiation trajectory inference

To model the cell state transition, the diffusion map algorithm, which preserves the global relations and pseudotemporal ordering of cells, is applied to infer the differentiation trajectory. We feed the attention matrix and the previously calculated principal components matrix into the scanpy pipeline. A neighborhood graph based on principal components is constructed using the scanpy.pp.neighbors function. The diffusion map is built using scanpy.tl.diffmap function. The first two diffusion components (DCs) are used for visualization. Partition-based graph abstraction (PAGA) analysis is also used for visualization. With the specifying of root cell, the diffusion pseudotime is calculated using scanpy.tl.dpt function.

To find the potential attentions driving the differentiation process, we fit a generalized additive model (gam function in the gam package of R) for the pseudotime and the attention matrix. Attentions with absolute coefficient >0.5 and FDR < 0.01 are considered as the dynamic attention terms.

### Statistics and reproducibility

No statistical method was used to predetermine sample size. Only data with poor labels were excluded from the analyses. The experiments were not randomized. The investigators were not blinded to allocation during experiments and outcome assessment.

### Reporting summary

Further information on research design is available in the Nature Portfolio Reporting Summary linked to this article.

## Supplementary information

Supplementary Information

Description of Additional Supplementary Files

Supplementary Dataset 1

Supplementary Dataset 2

Supplementary Dataset 3

Reporting Summary

## Data Availability

All datasets used are obtained from public data repositories. See Supplementary Dataset 1 for detailed information, including access codes. Tumor-infiltrating myeloid and T cells datasets are available from GEO “GSE154763” and “GSE156728”. COVID-19 and SLE datasets are available from GEO “GSE158055” and “GSE96583”. The mask matrix used in this work is based on knowledge datasets from “GSEA [http://www.gsea-msigdb.org/gsea/downloads.jsp]”. All other relevant data supporting the key findings of this study are available within the article or the Supplementary Information files. Source data are provided with this paper.

## Source data

Source Data

## Code availability

Software is available at “TOSICA [https://github.com/JackieHanLab/TOSICA]”^58^.
