# Supplementary material for: Transformer for one stop interpretable cell type annotation
Source: Nat Commun. 2023 Jan 14;14:223. doi: 10.1038/s41467-023-35923-4 (PMC9840170; doi:10.1038/s41467-023-35923-4)
Supplement: Supplementary file 2 — Description of Additional Supplementary Files [file 41467_2023_35923_MOESM2_ESM.pdf]

Descriptions of additional supplementary files

Supplementary Dataset 1. Meta information of datasets

Supplementary Dataset 2. Confusion matrix of TOSICA's prediction on mAtlas

Supplementary Dataset 3. Accuracy and rank of methods across datasets
