# Supplementary material for: Transformer for one stop interpretable cell type annotation
Source: Nat Commun. 2023 Jan 14;14:223. doi: 10.1038/s41467-023-35923-4 (PMC9840170; doi:10.1038/s41467-023-35923-4)
Supplement: Supplementary file 6 — Reporting Summary [file 41467_2023_35923_MOESM6_ESM.pdf]

nature portfolio

Corresponding author(s): Jing-Dong Jackie Han

Last updated by author(s): Dec 18, 2022

Reporting Summary

Nature Portfolio wishes to improve the reproducibility of the work that we publish. This form provides structure for consistency and transparency in reporting. For further information on Nature Portfolio policies, see our Editorial Policies and the Editorial Policy Checklist.

Statistics

For all statistical analyses, confirm that the following items are present in the figure legend, table legend, main text, or Methods section.

n/a	Confirmed	
☐	☒	The exact sample size (*n*) for each experimental group/condition, given as a discrete number and unit of measurement
☐	☒	A statement on whether measurements were taken from distinct samples or whether the same sample was measured repeatedly
☐	☒	The statistical test(s) used AND whether they are one- or two-sided<br>*Only common tests should be described solely by name; describe more complex techniques in the Methods section.*
☐	☒	A description of all covariates tested
☐	☒	A description of any assumptions or corrections, such as tests of normality and adjustment for multiple comparisons
☐	☒	A full description of the statistical parameters including central tendency (e.g. means) or other basic estimates (e.g. regression coefficient) AND variation (e.g. standard deviation) or associated estimates of uncertainty (e.g. confidence intervals)
☒	☐	For null hypothesis testing, the test statistic (e.g. *F*, *t*, *r*) with confidence intervals, effect sizes, degrees of freedom and *P* value noted<br>*Give P values as exact values whenever suitable.*
☒	☐	For Bayesian analysis, information on the choice of priors and Markov chain Monte Carlo settings
☒	☐	For hierarchical and complex designs, identification of the appropriate level for tests and full reporting of outcomes
☒	☐	Estimates of effect sizes (e.g. Cohen's *d*, Pearson's *r*), indicating how they were calculated

Our web collection on statistics for biologists contains articles on many of the points above.

Software and code

Policy information about availability of computer code

Data collection	Human pancreas datasets with cell type labels are downloaded using R package 'LTLA/scRNAseq' (version 2.7.2). Other datasets are collected without software but with access codes.
Data analysis	The codes of TOSICA for one-stop interpretable cell-type annotation can be downloaded at https://github.com/JackieHanLab/TOSICA<br>Python version 3.8.11 and R version 4.0.5 were used for downstream analysis with the following packages: torch (version 1.7.1), scanpy (version 1.7.1), Seurat (version 4.1.0), ggplot2 (3.3.5), ComplexHeatmap (2.10.0), gam (1.22) and their package dependencies. Benchmarking is done with scib (1.0.0).

For manuscripts utilizing custom algorithms or software that are central to the research but not yet described in published literature, software must be made available to editors and reviewers. We strongly encourage code deposition in a community repository (e.g. GitHub). See the Nature Portfolio guidelines for submitting code & software for further information.

Data

Policy information about availability of data

All manuscripts must include a data availability statement. This statement should provide the following information, where applicable:

- Accession codes, unique identifiers, or web links for publicly available datasets
- A description of any restrictions on data availability
- For clinical datasets or third party data, please ensure that the statement adheres to our policy

All datasets used in this study are already published and are obtained from public data repositories. See Supplementary Data 1 for detailed information, including access codes. The mask matrix used in this work is based on knowledge datasets from GSEA (http://www.gsea-msigdb.org/gsea/downloads.jsp). Source data are provided with this paper.

Human research participants

Policy information about studies involving human research participants and Sex and Gender in Research.

Reporting on sex and gender	Our study are lack of sex or gender information because we do not recruit participants by ourselves.
Population characteristics	We do not recruit participants by ourselves.
Recruitment	We do not recruit participants by ourselves.
Ethics oversight	We do not recruit participants by ourselves.

Note that full information on the approval of the study protocol must also be provided in the manuscript.

Field-specific reporting

Please select the one below that is the best fit for your research. If you are not sure, read the appropriate sections before making your selection.

☒ Life sciences ☐ Behavioural & social sciences ☐ Ecological, evolutionary & environmental sciences

For a reference copy of the document with all sections, see nature.com/documents/nr-reporting-summary-flat.pdf

Life sciences study design

All studies must disclose on these points even when the disclosure is negative.

Sample size	We use 6 demo datasets to do the benchmark. They are all common datasets to judge the goodness of methods. Two real world example datasets includes hundred thousands data to verify our method's scalability.
Data exclusions	Cells with bad annotation are excluded.
Replication	We fixed the random seed during training for reproducibility, and other default hyperparameters are provided. For other methods we use the default parameters.
Randomization	We divide the datasets as training and test set as mentioned in Supplementary table 1's meta information of datasets or in the text.
Blinding	Blinding is not applicable as all the data we use is published data with clear labels

Reporting for specific materials, systems and methods

We require information from authors about some types of materials, experimental systems and methods used in many studies. Here, indicate whether each material, system or method listed is relevant to your study. If you are not sure if a list item applies to your research, read the appropriate section before selecting a response.

Materials & experimental systems

n/a	Involved in the study
☒	☐ Antibodies
☒	☐ Eukaryotic cell lines
☒	☐ Palaeontology and archaeology
☒	☐ Animals and other organisms
☒	☐ Clinical data
☒	☐ Dual use research of concern

Methods

n/a	Involved in the study
☒	☐ ChIP-seq
☒	☐ Flow cytometry
☒	☐ MRI-based neuroimaging
