# Supplementary material for: Transformer for one stop interpretable cell type annotation
Source: Nat Commun. 2023 Jan 14;14:223. doi: 10.1038/s41467-023-35923-4 (PMC9840170; doi:10.1038/s41467-023-35923-4)
Supplement: Supplementary file 1 — Supplementary Information [file 41467_2023_35923_MOESM1_ESM.pdf]

Supplementary Information

Transformer for One Stop Interpretable Cell type Annotation

Jiawei Chen [1*], Hao Xu[1*], Wanyu Tao[1], Zhaoxiong Chen[1], Yuxuan Zhao[1] and Jing-Dong J. Han[1#]

[1] Peking-Tsinghua Center for Life Sciences, Academy for Advanced Interdisciplinary Studies, Center for Quantitative Biology (CQB), Peking University, Beijing 100871, China

[*] These authors contributed equally to this work

[#] Correspondence and requests for materials should be addressed to J.-D.J.H. (Email: jackie.han@pku.edu.cn)

Supplementary Figure 1

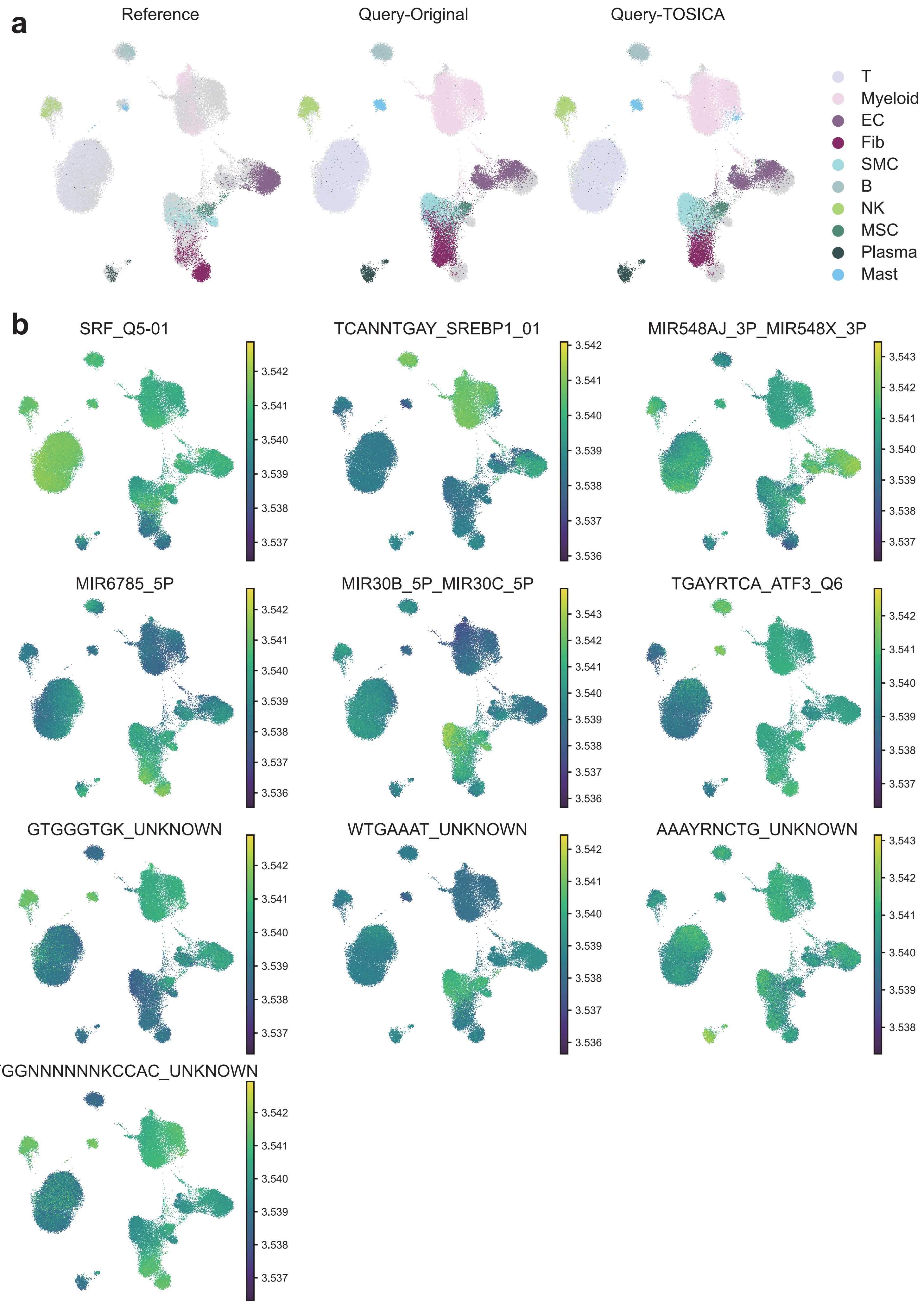

Supplementary Figure 1. TOSICA embeds, annotates hArtery dataset, and shows the important pathways for each cell type. a. UMAP representation of hArtery colored by cell types showing TOSICA matches cells in query (state = disease) to reference (state = healthy) and annotates them correctly. Gray means cells in test set (right), or cells in training set (middle and left). Prediction and embedding are done by TOSICA. **b.** UMAP representation of hArtery colored by attention scores showing the pathways most important for separating cell types by TOSICA.

Supplementary Figure 2

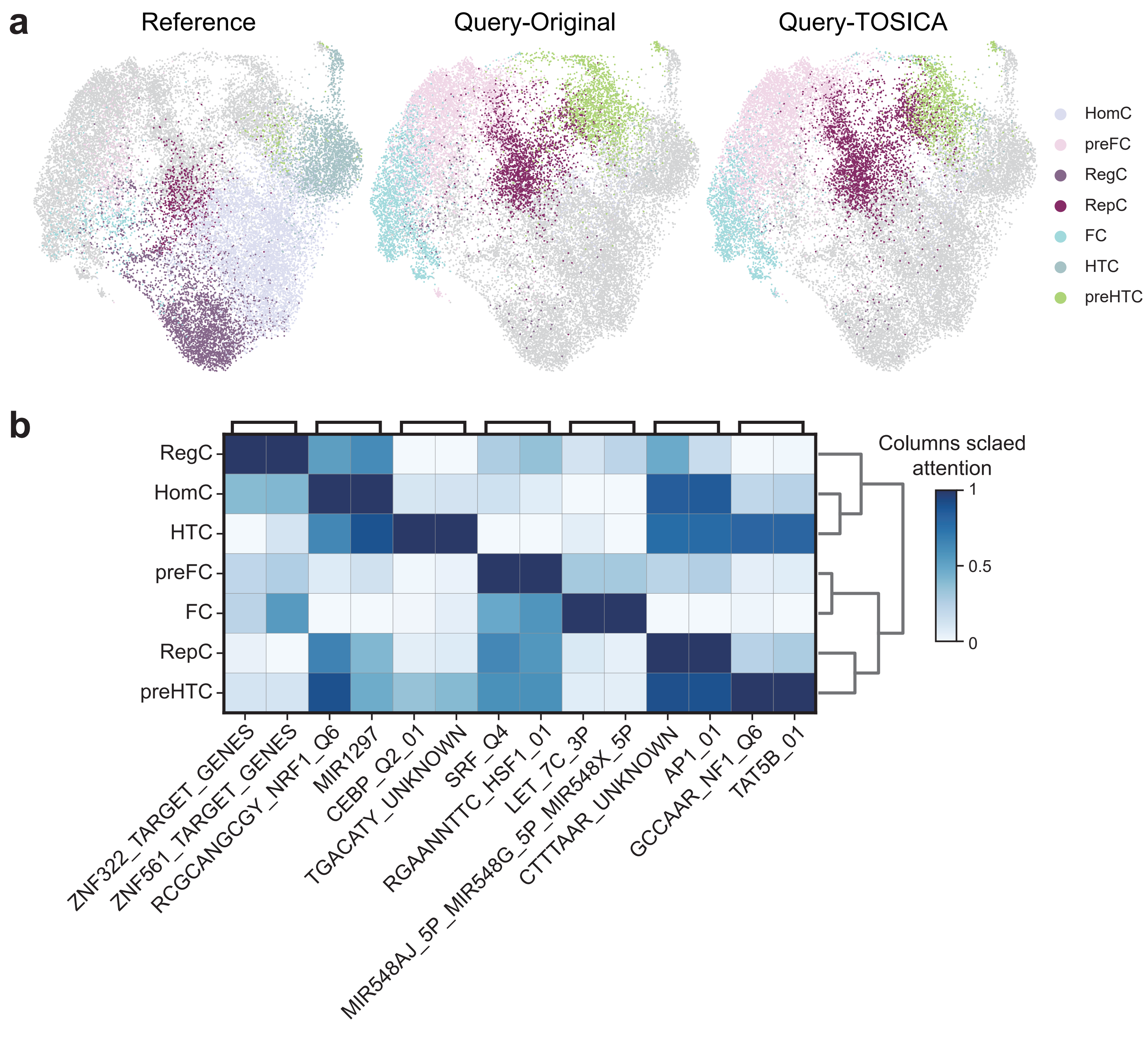

Supplementary Figure 2. TOSICA embeds, annotates hBone dataset, and shows the important pathways for each cell type. a. UMAP representation of hBone colored by cell types showing that TOSICA matches cells in query (disease sites, medial tibiae (MT)) to reference (healthy sites, outer lateral tibiae (OLT)) and annotates them correctly. Gray means cells in test set (right), or cells in training set (middle and left). Prediction and embedding are done by TOSICA. **b.** The pathways shown here are the most important ones to separate cell types by TOSICA.

Supplementary Figure 3

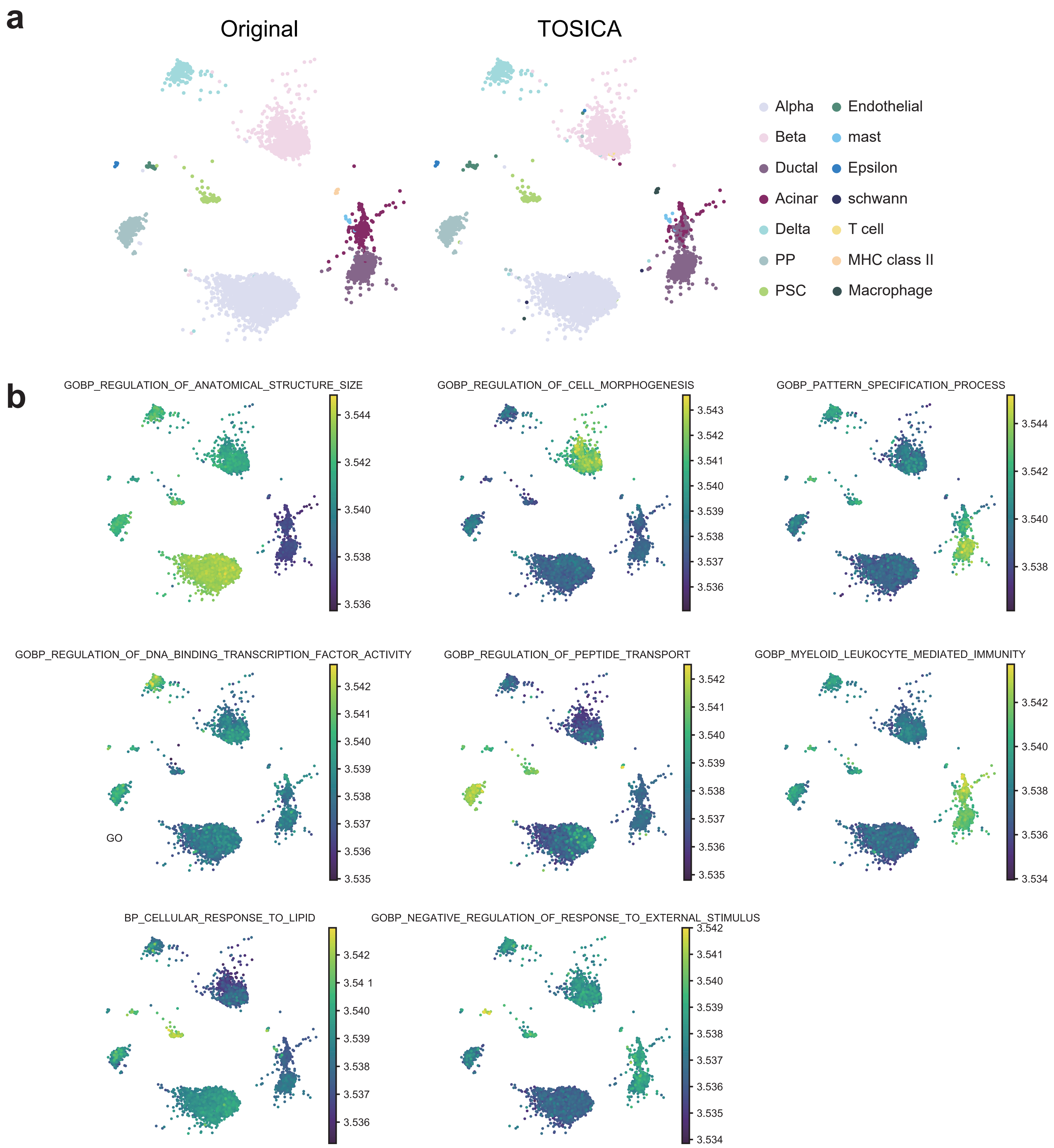

Supplementary Figure 3. TOSICA embeds, annotates hPancreas dataset, and shows the important pathways for each cell type. a. UMAP representation of hPancreas query set (study = xin, segerstolpe, Lawlor) colored by cell types showing accurate annotation of TOSICA, when using cells from Baron's and Muraro's studies as reference (not shown). Prediction and embedding are done by TOSICA. **b.** UMAP representation of hPancreas colored by attention scores showing the specific pathway identified by TOSICA that belongs to each cell type.

Supplementary Figure 4

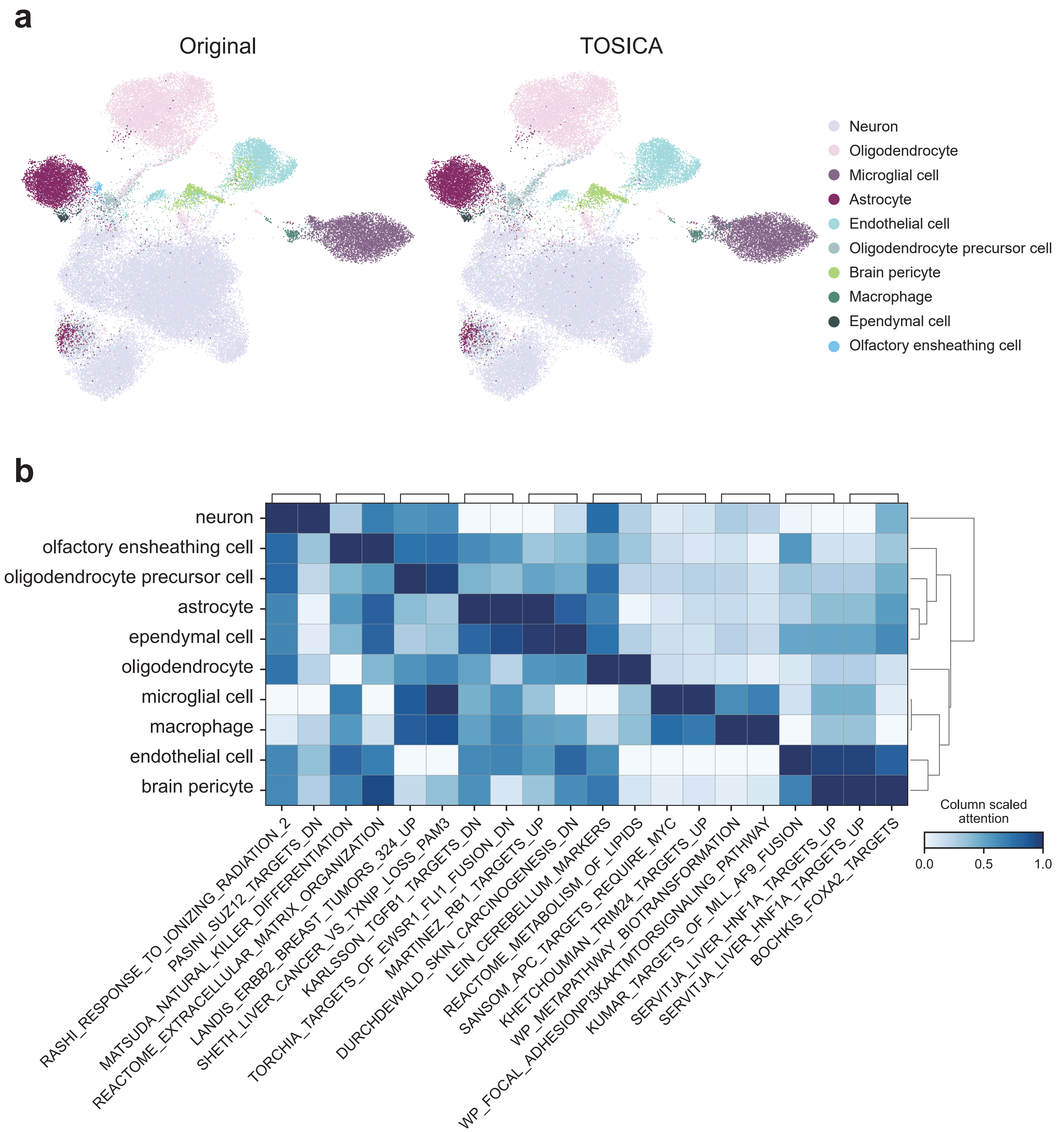

Supplementary Figure 4. TOSICA embeds, annotates mBrain dataset, and shows the important pathways for each cell type. a. UMAP representation of mBrain query set (study = Zeisel) colored by cell types showing TOSICA accurate annotation, when using cells from Saunders's, Tabula muris consortium's, and Rosenberg's studies as reference (not shown). **b.** The pathways shown here are the most important ones to separate cell types by TOSICA.

Supplementary Figure 5

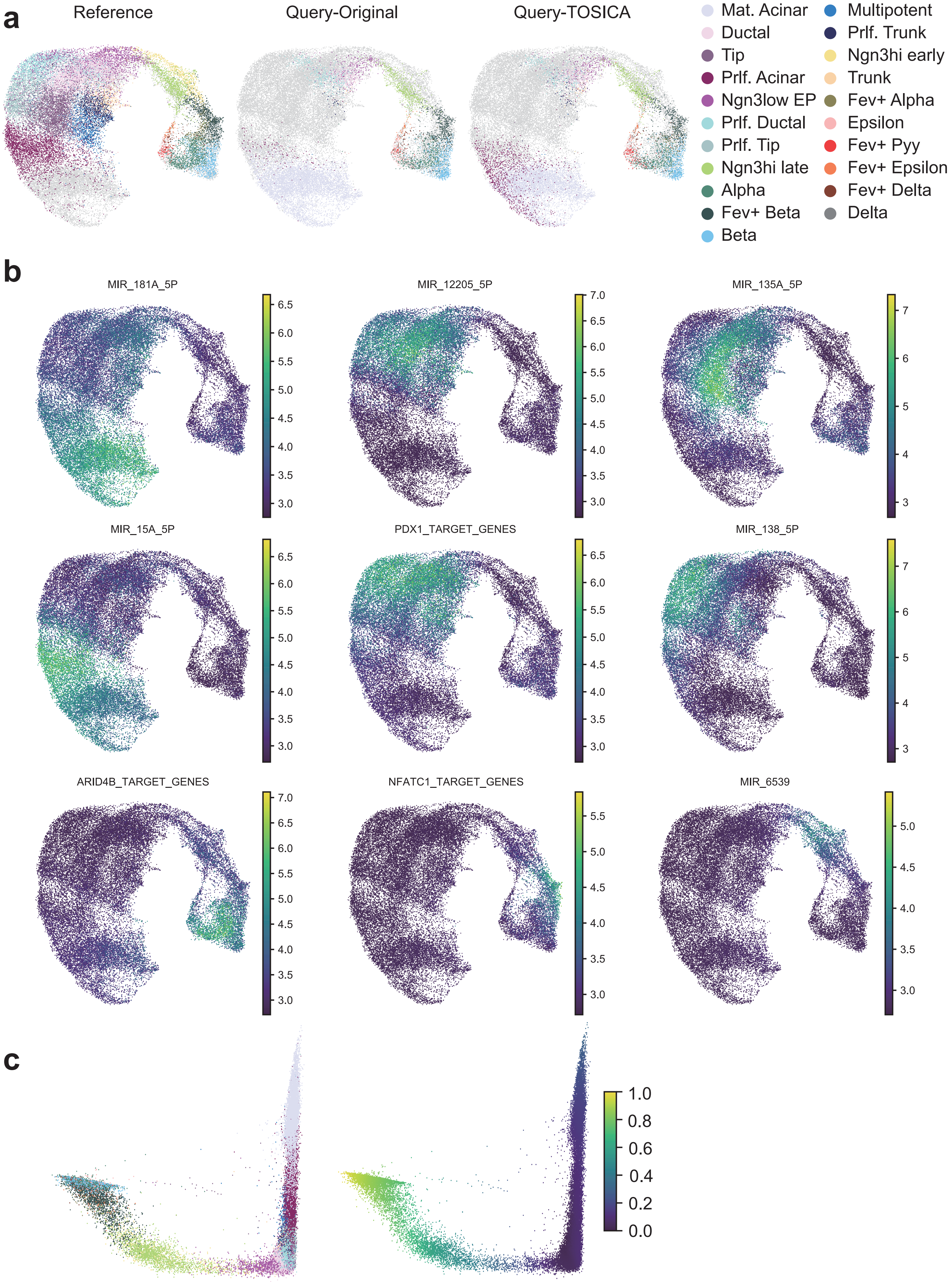

Supplementary Figure 5. TOSICA embeds, annotates mPancreas dataset, and shows the important pathways for each cell type. a. TOSICA succeeds in matching cells in query (mouse age = 15.5 day) to reference (mouse age $\neq$ 15.5 day) of mPancreas. UMAP representation of mPancreas is colored by cell types. Gray means cells in test set (right), or cells in training set (middle and left). Prediction and embedding are done by TOSICA. **b.** UMAP representation of mPancreas colored by attention scores showing the specific pathway that belongs to each cell type. **c.** Diffusion map representation of mPancreas colored by cell types (left) and pseudotime (right) showing the change in cell types during of pancreas development.

Supplementary Figure 6

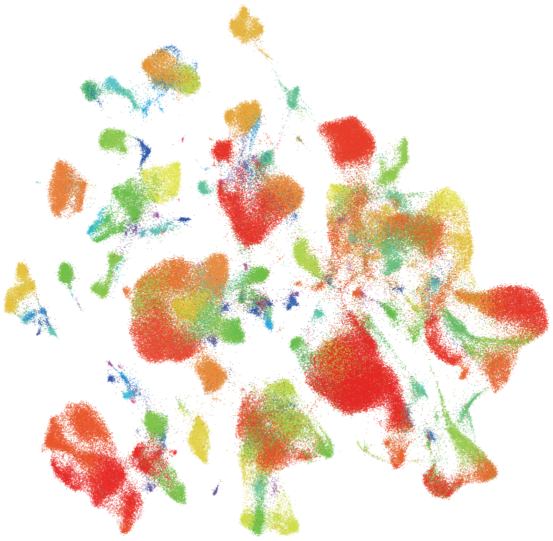

- B cell
- basal cell of epidermis
- granulocyte
- endothelial cell
- mesenchymal stem cell
- microglial cell
- T cell
- keratinocyte
- macrophage
- classical monocyte
- granulocytopoietic cell
- bladder urothelial cell
- mesenchymal stem cell of adipose
- fibroblast of cardiac tissue
- endothelial cell of coronary artery
- skeletal muscle satellite cell
- proerythroblast
- kidney proximal convoluted tubule epithelial cell
- bronchial smooth muscle cell
- myeloid cell
- hepatocyte
- pancreatic B cell
- bladder cell
- promonocyte
- DN4 thymocyte
- basal cell
- monocyte
- enterocyte of epithelium of large intestine
- thymocyte
- epithelial cell of proximal tubule
- NK cell
- naive B cell
- hematopoietic stem cell
- mature NK T cell
- fibroblast
- stromal cell
- erythroblast
- Kupffer cell
- megakaryocyte-erythroid progenitor cell
- pancreatic acinar cell
- luminal epithelial cell of mammary gland
- professional antigen presenting cell
- bulge keratinocyte
- epidermal cell
- hematopoietic precursor cell
- oligodendrocyte
- fibroblast of lung
- CD8-positive, alpha-beta T cell
- epithelial cell of large intestine
- intestinal crypt stem cell
- chondrocyte
- immature T cell
- precursor B cell
- intermediate monocyte
- large intestine goblet cell
- CD4-positive, alpha-beta T cell
- smooth muscle cell
- mesenchymal progenitor cell
- leukocyte
- plasma cell
- lymphocyte
- naive T cell
- kidney loop of Henle thick ascending limb epithelial cell
- immature B cell
- alveolar macrophage
- fenestrated cell
- endothelial cell of hepatic sinusoid
- lung macrophage
- non-classical monocyte
- endocardial cell
- double negative T cell
- kidney collecting duct principal cell
- late pro-B cell
- basal epithelial cell of tracheobronchial tree
- mesenchymal cell
- pancreatic A cell
- adventitial cell
- epithelial cell
- kidney distal convoluted tubule epithelial cell
- neutrophil
- myeloid dendritic cell
- astrocyte
- secretory cell
- myeloid leukocyte
- podocyte
- brain pericyte
- neuron
- vein endothelial cell
- kidney collecting duct epithelial cell
- aortic endothelial cell
- pancreatic D cell
- keratinocyte stem cell
- brush cell
- erythroid progenitor
- atrial myocyte
- kidney cortex artery cell
- erythrocyte
- kidney loop of Henle ascending limb epithelial cell
- pancreatic ductal cel
- cardiomyocyte
- mature alpha-beta T cell
- basophil
- valve cell
- pancreatic ductal cell
- oligodendrocyte precursor cell
- type II pneumocyte
- Schwann cell
- pulmonary interstitial fibroblast
- kidney mesangial cell
- smooth muscle cell of trachea
- granulocyte monocyte progenitor cell
- interneuron
- pancreatic PP cell
- smooth muscle cell of the pulmonary artery
- skeletal muscle cell
- Langerhans cell
- Brush cell of epithelium proper of large intestine
- stem cell of epidermis
- regulatory T cell
- epithelial cell of thymus
- enteroendocrine cell
- kidney capillary endothelial cell
- neuroepithelial cell
- macrophage dendritic cell progenitor
- ventricular myocyte
- DN3 thymocyte
- endothelial cell of lymphatic vessel
- ciliated columnar cell of tracheobronchial tree
- plasmacytoid dendritic cell
- dendritic cell
- medium spiny neuron
- pericyte cell
- neuronal stem cell
- kidney proximal straight tubule epithelial cell
- mesangial cell
- blood cell
- immature NKT cell
- cardiac neuron
- mast cell
- mucus secreting cell
- early pro-B cell
- pancreatic stellate cell
- ependymal cell
- Bergmann glial cell
- neuroendocrine cell
- fibrocyte
- hepatic stellate cell
- club cell of bronchiole
- respiratory basal cell
- kidney interstitial fibroblast
- lymphoid progenitor cell
- lung neuroendocrine cell
- type I pneumocyte
- kidney cell
- duct epithelial cell

Supplementary Figure 6. UMAP representation of the whole mAtlas colored by cell types, embedding is done with TOSICA attention scores.

Supplementary Figure 7

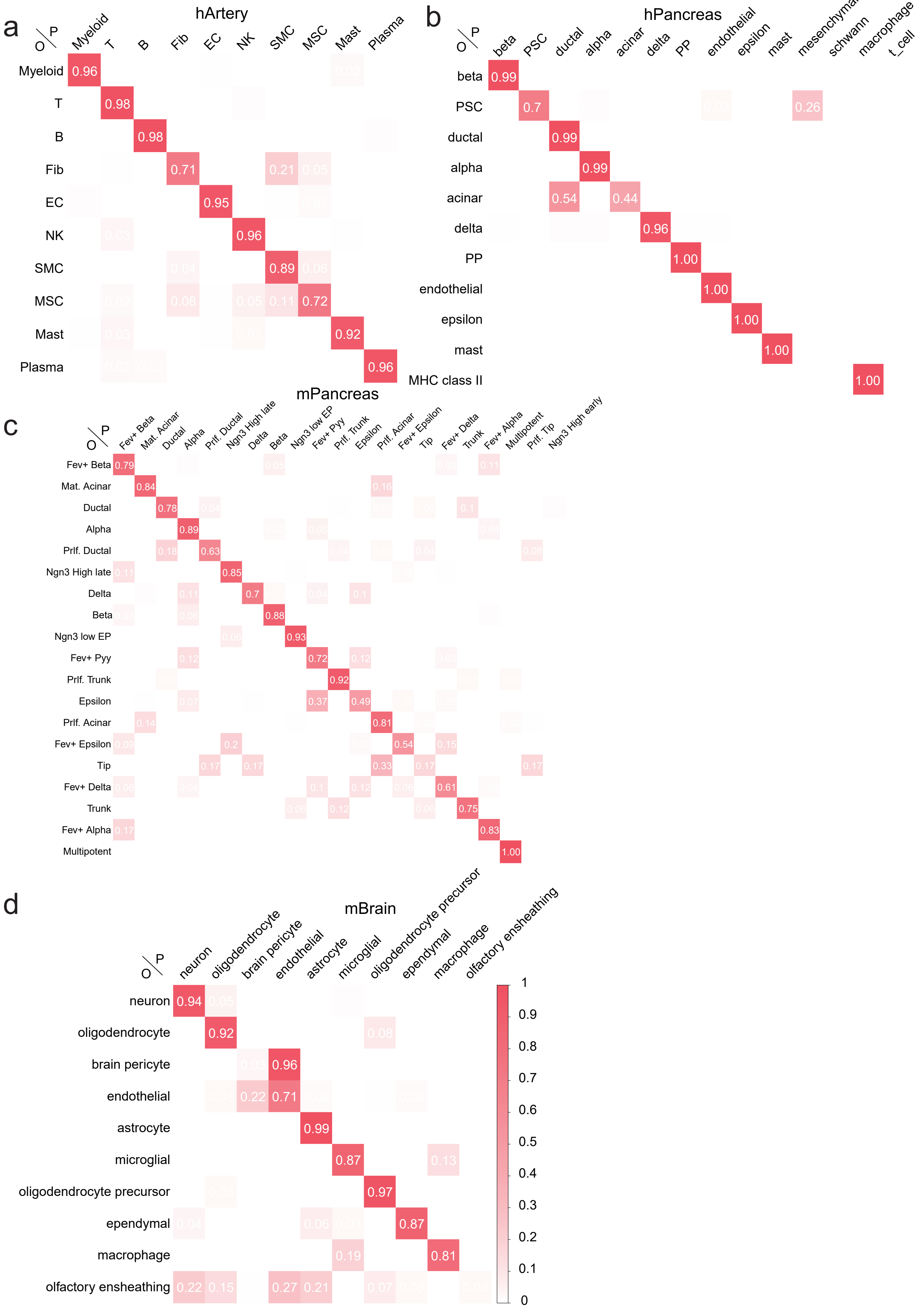

Supplementary Figure 7. Confusion matrix of TOSICA's prediction on hArtery (a), hPancreas (b), mPancreas (c) and mBrain (d). hBone's results has been shown in Fig. 2e, and mAtlas' results is in Supplementary Dataset 2. Source data are provided as a Source Data file.

Supplementary Figure 8

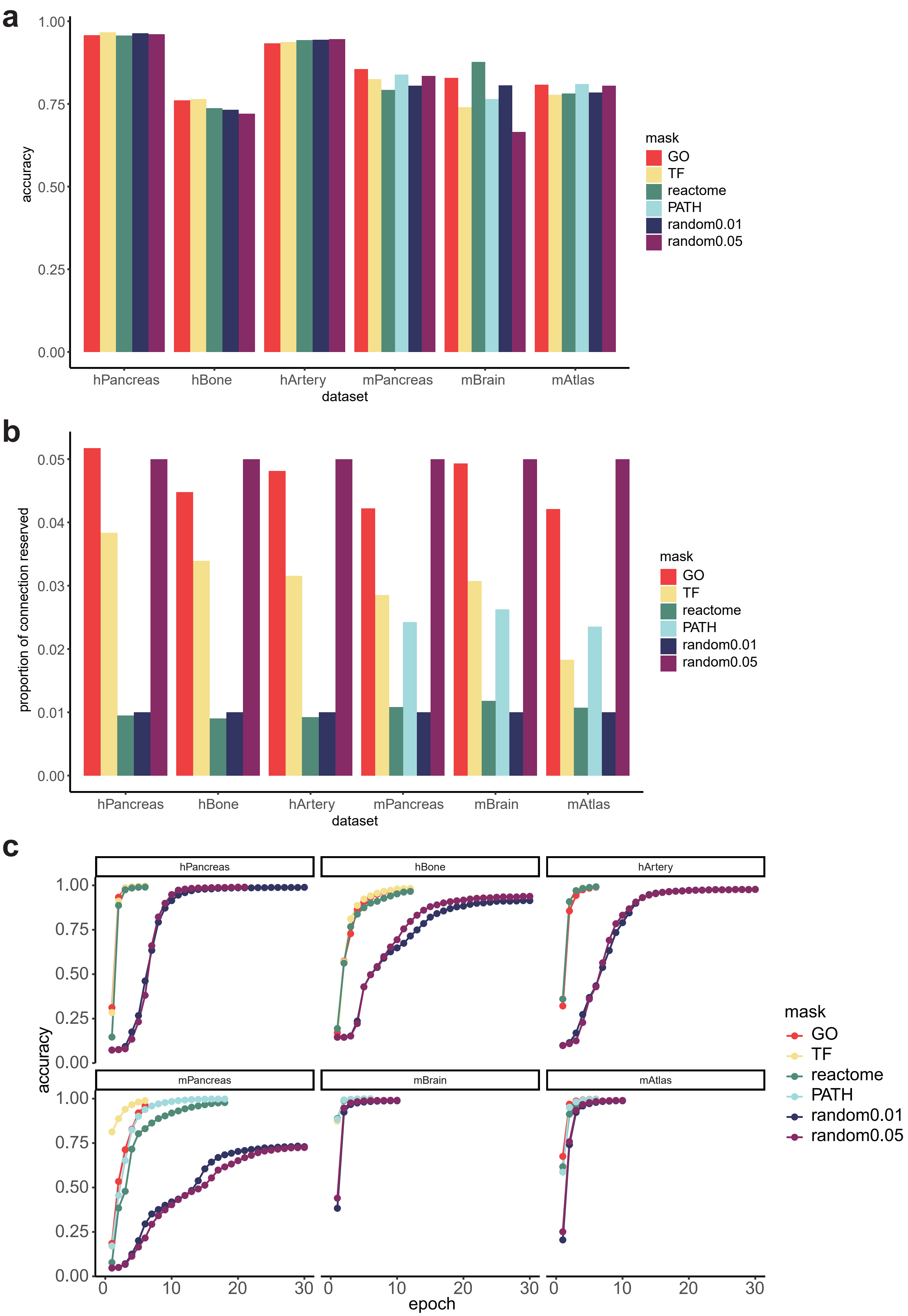

Supplementary Figure 8. Impact of different masks on TOSICA. a. Choice of mask does not affect TOSICA's accuracy on query dataset. **b.** The proportion of connections reserved in real world varies from 0.01 to 0.05. **c.** Comparing with expert knowledge-based mask, model using random mask with same proportion of connections reserved takes more epochs to converge when training. Source data are provided as a Source Data file.

Supplementary Figure 9

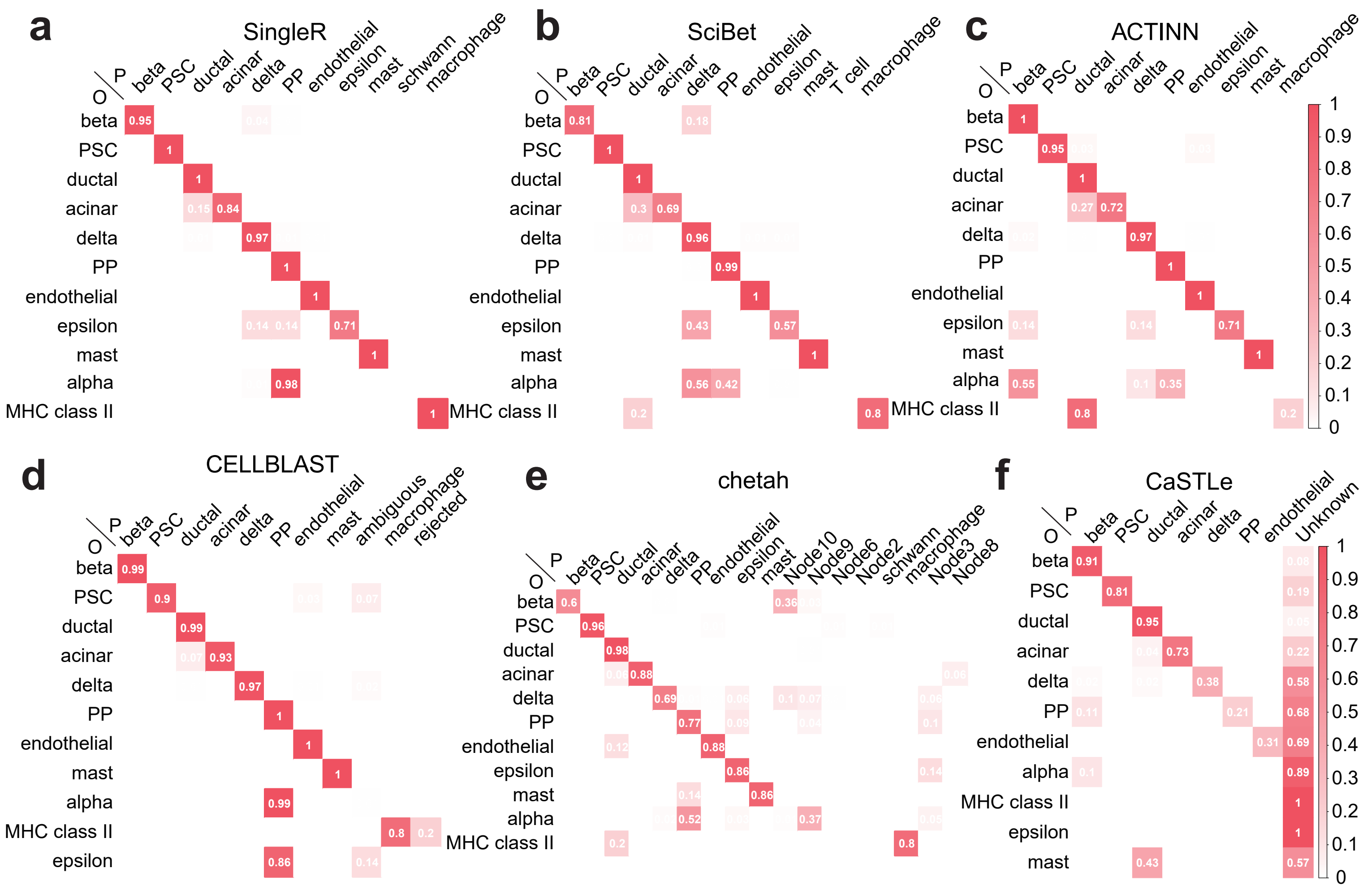

Supplementary Figure 9. TOSICA performs better than six other methods to label masked alpha cell as 'unknown' in hPancreas. SingleR, Scibet and ACTINN are next 3 top mean accuracy methods. CELLBLAST, chetah, CaSTLe are the next 3 top mean accuracy methods that are able to discover new cell types. TOSICA's performance is shown in Fig. 3b. Mean accuracies of different methods are shown in Fig. 3a, and decrease sequentially from panels a to f. Source data are provided as a Source Data file.

Supplementary Figure 10

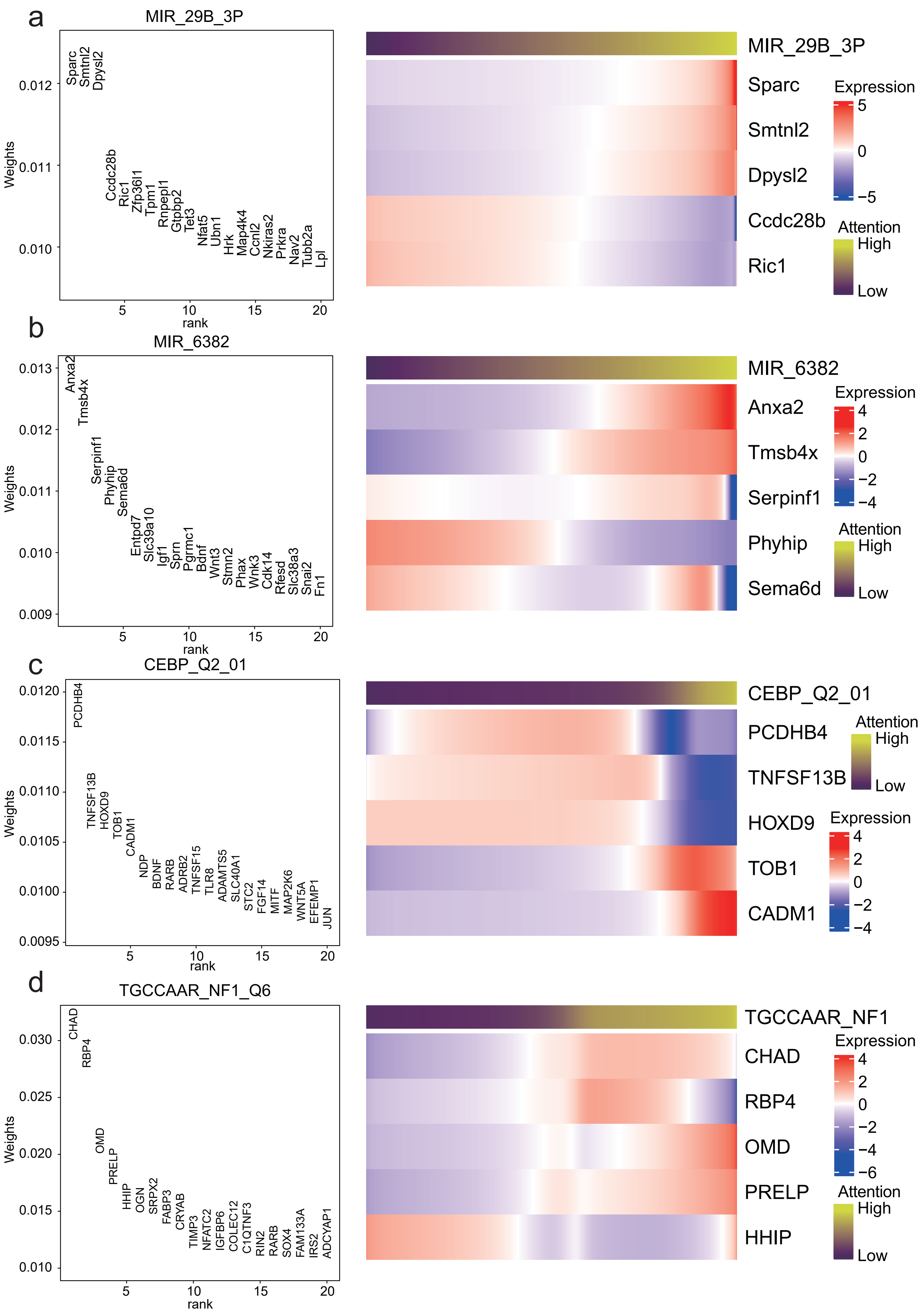

Supplementary Figure 10. Top 20 important genes by TOSICA weights for MIR_29B_3P (a), MIR_6382 (b), CEBP_Q2_01 (c) and TGCCAAR_NF1_Q6 (d) regulons, and smoothed expression level of top 5 important genes in each single cell, sorted by attention score.

Supplementary Figure 11

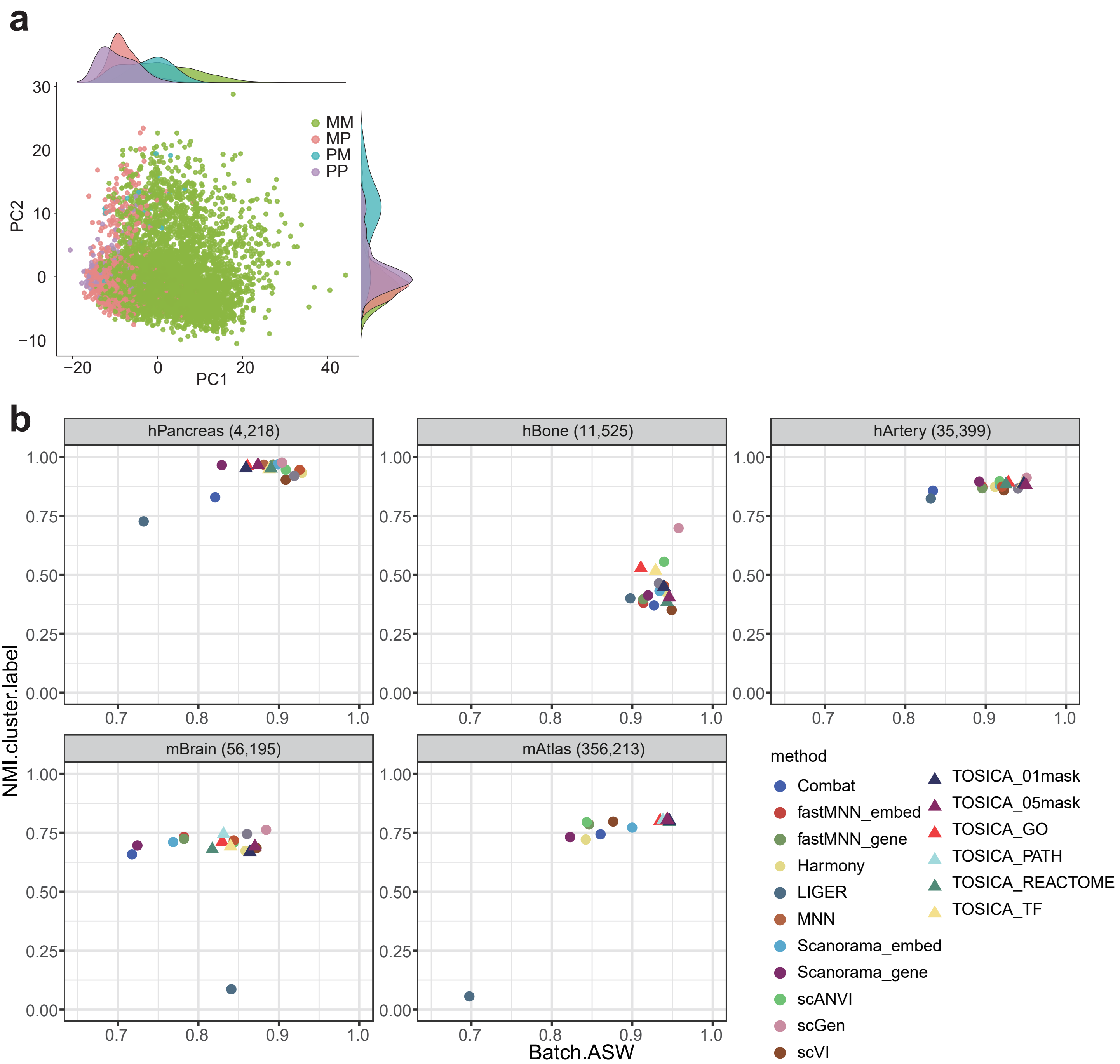

Supplementary Figure 11. PCA analyses of acinar cells in mPancreas dataset, and comparison of TOSICA with other methods on batch effect removal. a. Principal component analysis of the original expression matrix also shows that MP shares similar PCs with PP (reference Prlf. Acinar, TOSICA Prlf. Acinar) compared with other MM, where the transition ordering is visible on PC1 **b.** TOSICA is insensitive to batch effect especially on large datasets. The NMI cluster label and Batch ASW score of TOSICA and other methods are shown here. The size of each dataset is shown as number of cells inside the parenthesis in the dataset label. In respective with inter-method differences, TOSICA when using different masks does not differ much in batch effect removal and biological conservation shown by both ASW and NMI on most datasets, except batch effects removal ability is unexpectedly slightly stronger when using random masks, and as expected some knowledge based masks are better than others for a specific dataset. Source data are provided as a Source Data file.

Supplementary Figure 12

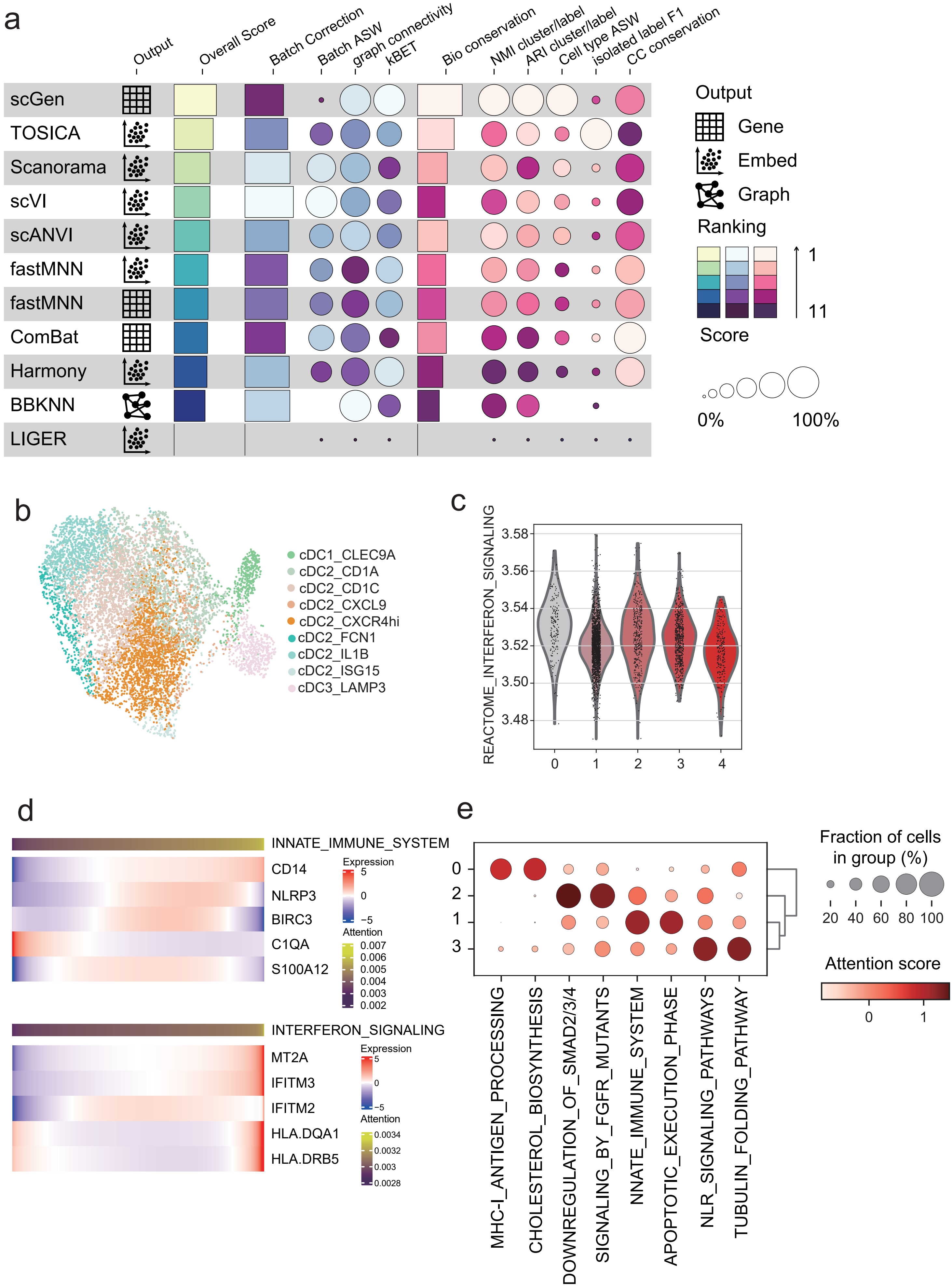

Supplementary Figure 12. TOSICA resolves pan-cancer tumor infiltrating myeloid cell heterogeneity. a. Comparison of integration accuracy on query (same as Fig. 4a) data of pan-cancer tumor infiltrating myeloid cells across various methods ranks TOSICA the second out of 11 methods. Individual scores are minimum–maximum scaled between 0 and 1. Overall scores are computed as a 40:60-weighted mean of batch correction and bio-conservation scores. **b.** TOSICA attention based UMAP of cDCs colored by subtypes. **c.** The attention score of INTERFERON_SIGNALINGS decreases with advanced stage of ESCA. **d.** The smoothed expression level of top 5 important genes by TOSICA weight for INNATE_IMMUNE_SYSTEM (up) and INTERFERON_SIGNALING (down) is consistent with the attention trend, including NLRP3 and IFITM3, sorted by attention score. **e.** REACTOME pathways distinguish 4 subtypes of monocyte by TOSICA. Source data are provided as a Source Data file.

Supplementary Figure 13

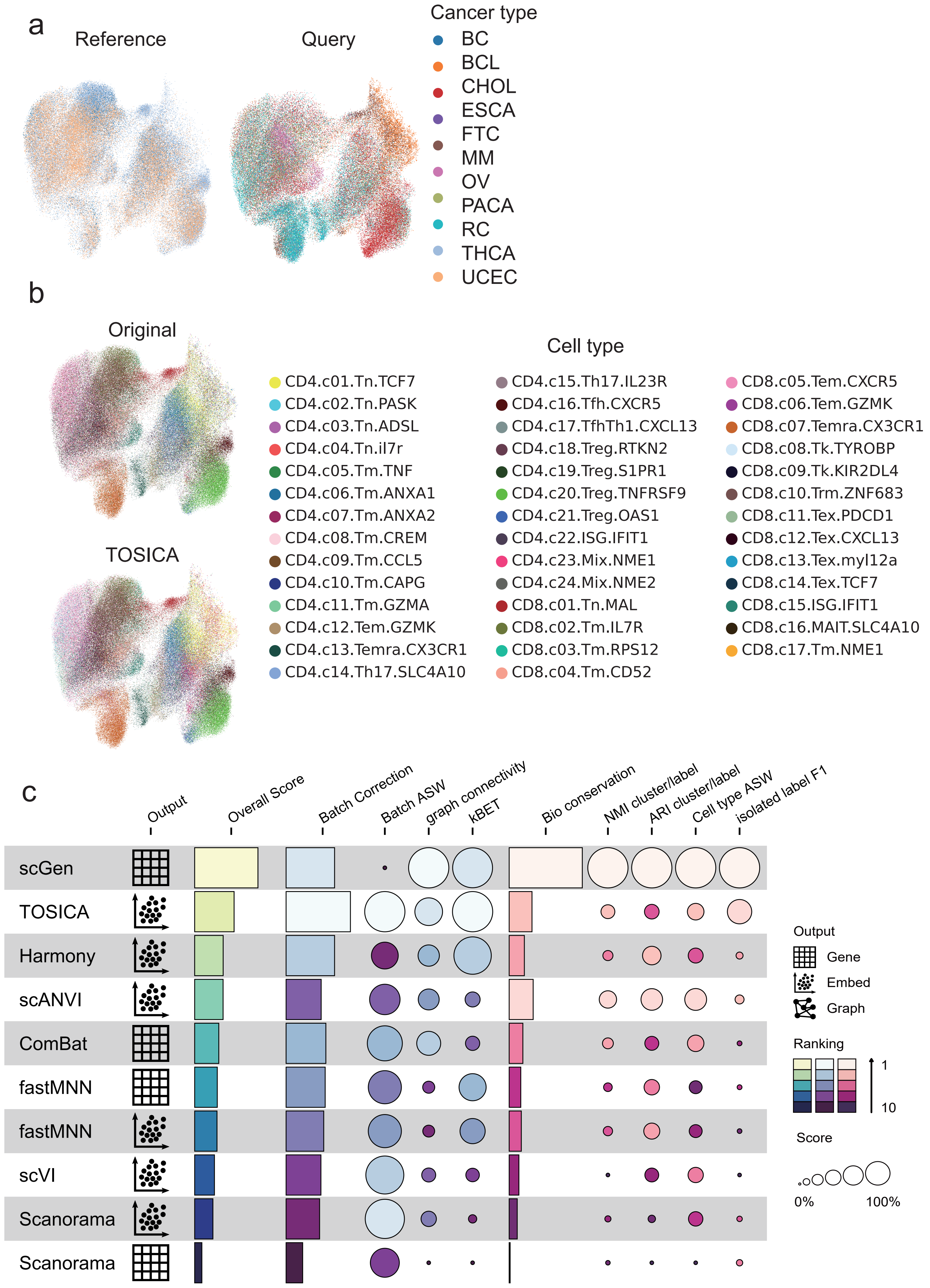

Supplementary Figure 13. TOSICA integrates and annotates pan-cancer tumor infiltrating T cell heterogeneity. a. TOSICA predicts cell types reliably across different cell types even when the reference and query contain no overlapping cancer types. TOSICA attention embedded UMAP is colored by the cancer types in the reference (n=3) and query (n=7), **b.** and by cell types in the query as originally labeled and predicted by TOSICA. **c.** Comparison of integration accuracy on query data of pan-cancer tumor infiltrating T cells across various methods. Individual scores are minimum–maximum scaled between 0 and 1. Overall scores are computed as a 40:60-weighted mean of batch correction and bio-conservation scores. Source data are provided as a Source Data file.

Supplementary Figure 14

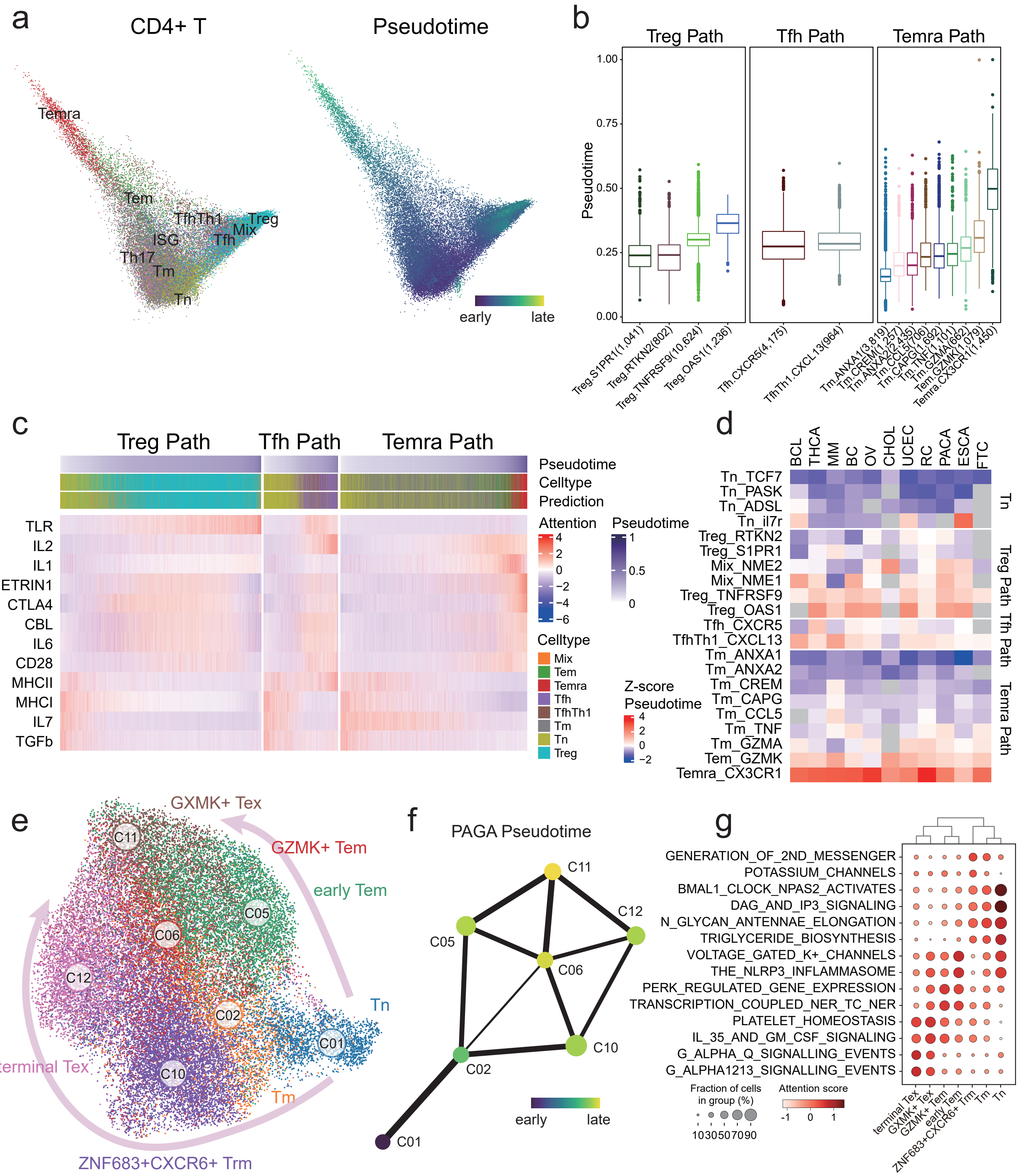

Supplementary Figure 14. TOSICA resolves pan-cancer tumor infiltrating T cell heterogeneity. a. Three developmental paths from Tn to Treg, Tfh and Temra are delineated on diffusion map of CD4+ T on TOSICA attention embedding. Colors denote major cell types (left) and pseudotime (right). **b.** Sub-cell types show continuous pseudotime change in each state transition path, sorted by median pseudotime in each cell types. Cell numbers are annotated behind cell types. Boxes depict the median values and upper and lower quartiles of the data, and whiskers depict the range of the data excluding outliers (outliers are defined as data-points > 1.5 × the inter-quartile range from the upper or lower quartiles). **c.** The dominant TOSICA attention pathways are different in each path, showing differential attention scores in each single cell, sorted by state transition path and pseudotime. Pseudotime, origin labels and prediction labels are shown on the top. **d.** CD4+ T shows heterogeneity of dynamic in different cancers. Source data are provided as a Source Data file. **e.** TOSICA attention embedded UMAP showing two major state transition paths, Tem path and Trm path, from naïve to exhaustion. Dots are colored by major cell types. **f.** PAGA reconstruction of the state transition paths. Edge weights represent confidence in the presence of connections between cell types colored by pseudotime. **g.** REACTOME pathways identified by TOSICA separate the subtypes of CD8+ T cells.

Supplementary Figure 15

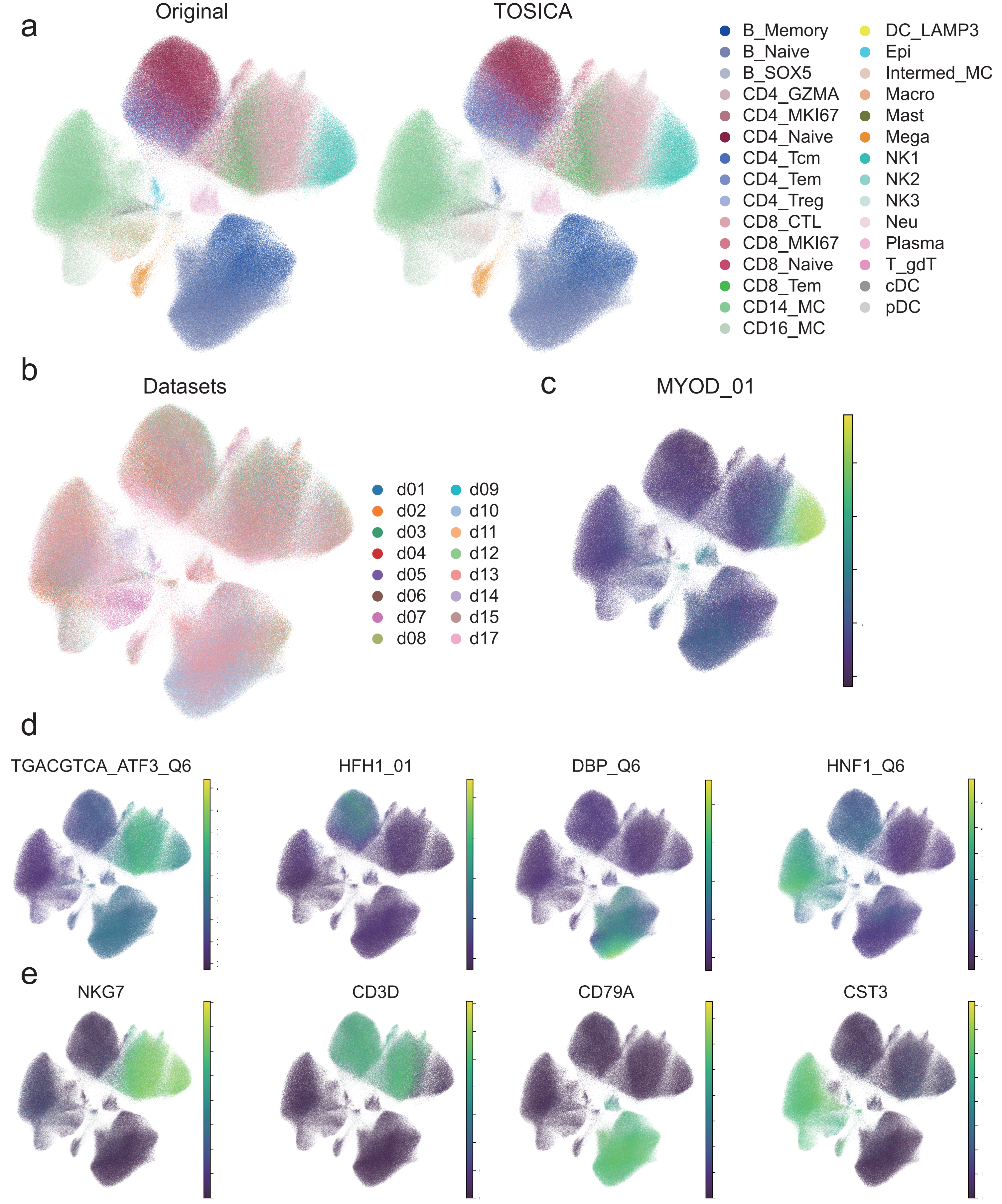

Supplementary Figure 15. TOSICA embeds cells, predicts cell types and identifies TF regulons in COVID19 dataset with no batch effect. a, b. All cells from reference and query are predicted by TOSICA and visualized by TOSICA attention embedded UMAP. Colors denote the original labels (panel a left), TOSICA predicted label (panel a right) and datasets (panel b). **c, d.** Attention score of TF regulons identified by TOSICA to separate NK (c) and other (d) cell types are shown on UMAP. MYOD regulon is used a classical marker TF of NK for comparison. **e.** UMAP showing the expression levels of the classical marker genes in different cell types.

Supplementary Figure 16

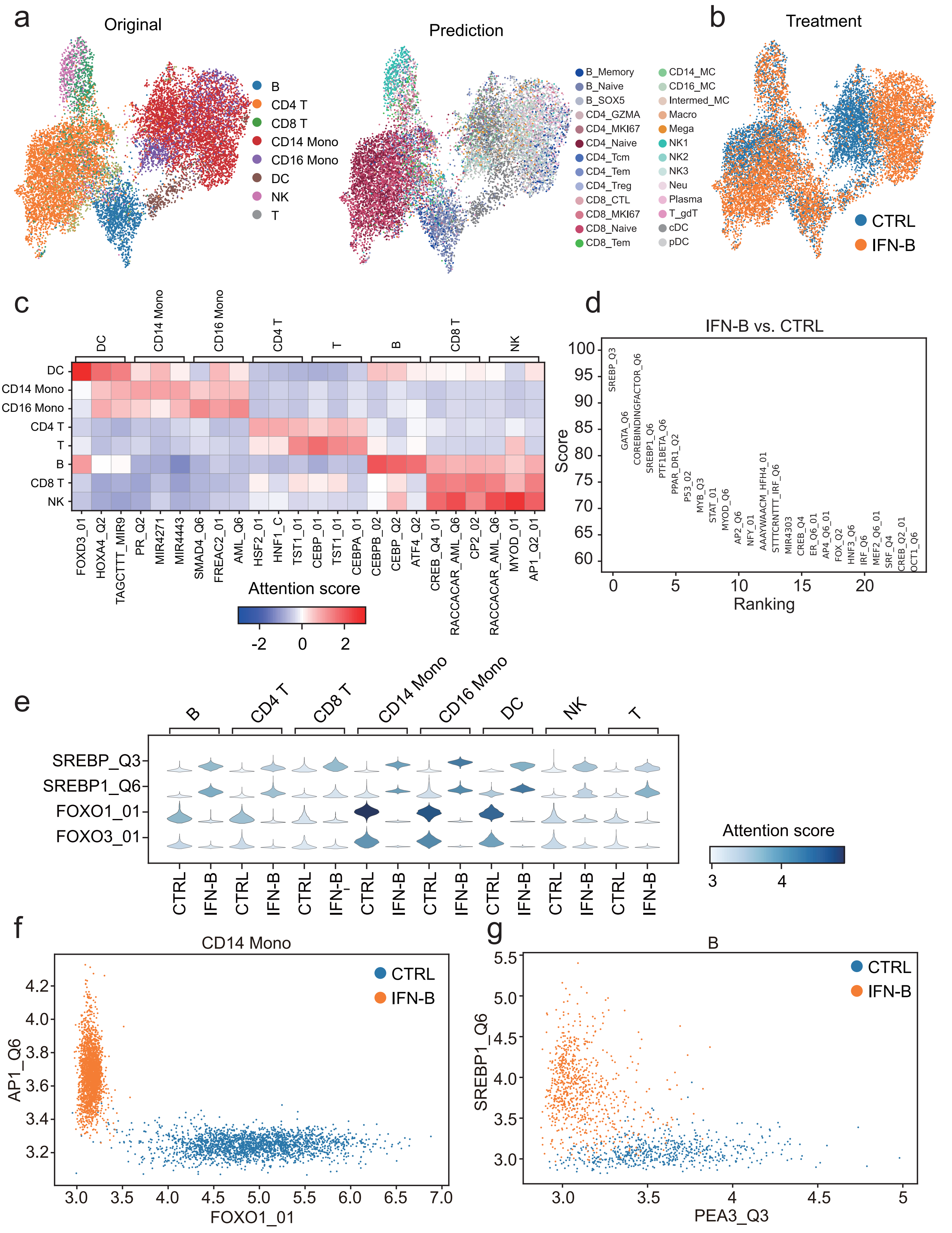

Supplementary Figure 16. TOSICA transfers cell type labels from COVID19 dataset to SLE dataset and reveals the effect of IFN-β treatment. a, b, TOSICA predicts cell types reliably across different cell types even when the reference (COVID19 datasets) and query (SLE) come from different disease datasets. TOSICA attention embedded UMAP distinguishes the untreated and IFN-β treated PBMC of SLE patients. Colors denote original label or predicted label (a.) and treatment (b.). **c.** Heatmap of TOSICA attention TF regulons of major cell types. **d.** Differential attention scores of TF regulons between IFN-β and control cells. The x-axis shows the ranking of TF regulons; the y-axis denotes the significance (absolute log-Bayes factor) of each regulon. **e.** The activity of the most differentially active TF regulon (SREBP/SREBP1 up-regulated and FOXO1/3 down-regulated) in major cell types after IFN-β stimulation. Violin plots display the distribution of attention score across different cell types. **f, g.** TOSICA gives different attention scores on the two pairs of TF regulons for CD14 Mono (f) and B (g) cells under IFN-β stimulation versus untreated cells.

Supplementary Figure 17

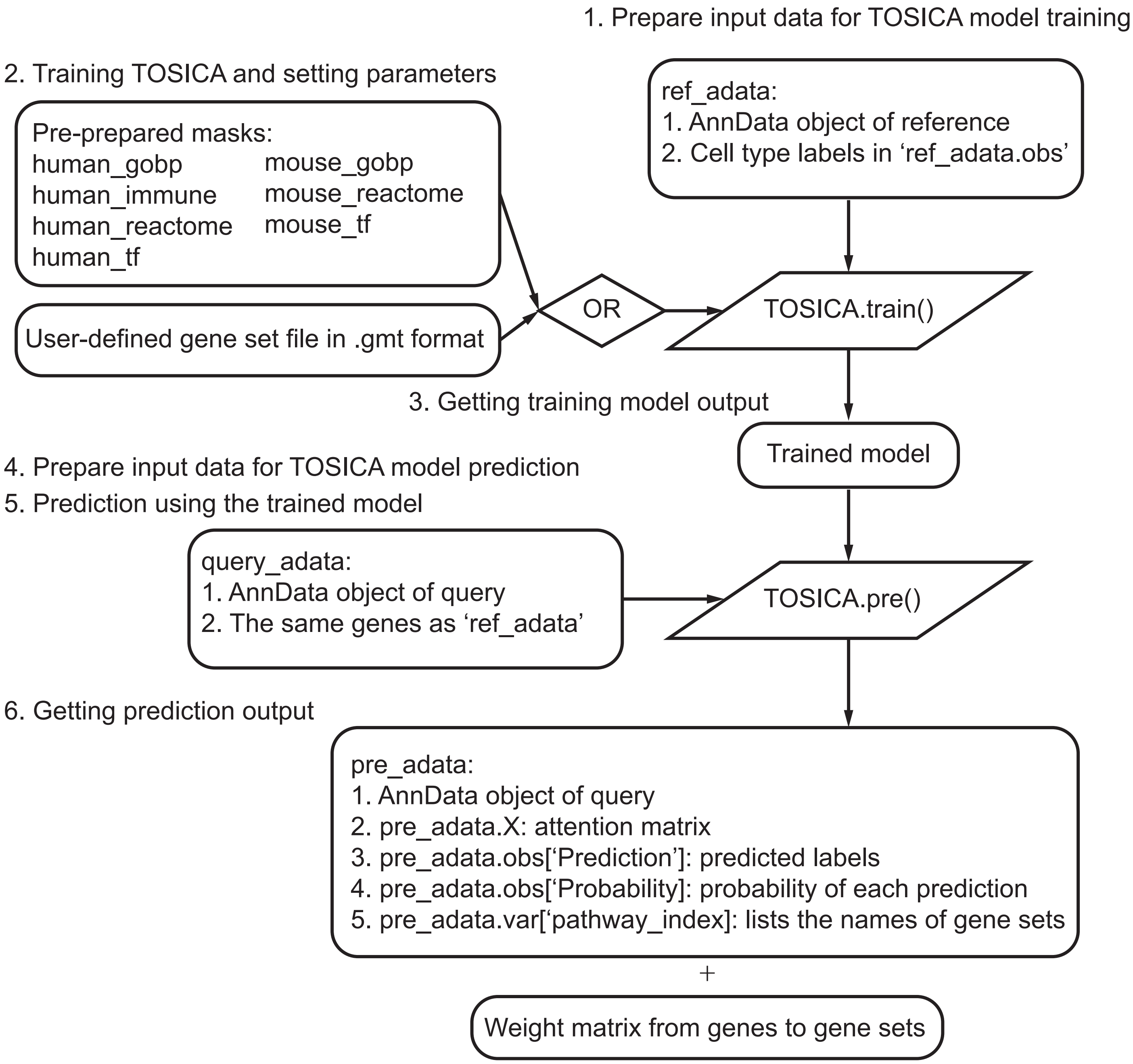

Supplementary Figure 17. Workflow schematic of using TOSICA. Using TOSICA includes the following steps: 1. Prepare input data for TOSICA model training; 2. Training TOSICA and setting parameters; 3. Getting training model output; 4. Prepare input data for TOSICA model prediction; 5. Prediction using the trained model; 6. Getting prediction output.

Supplementary Figure 18

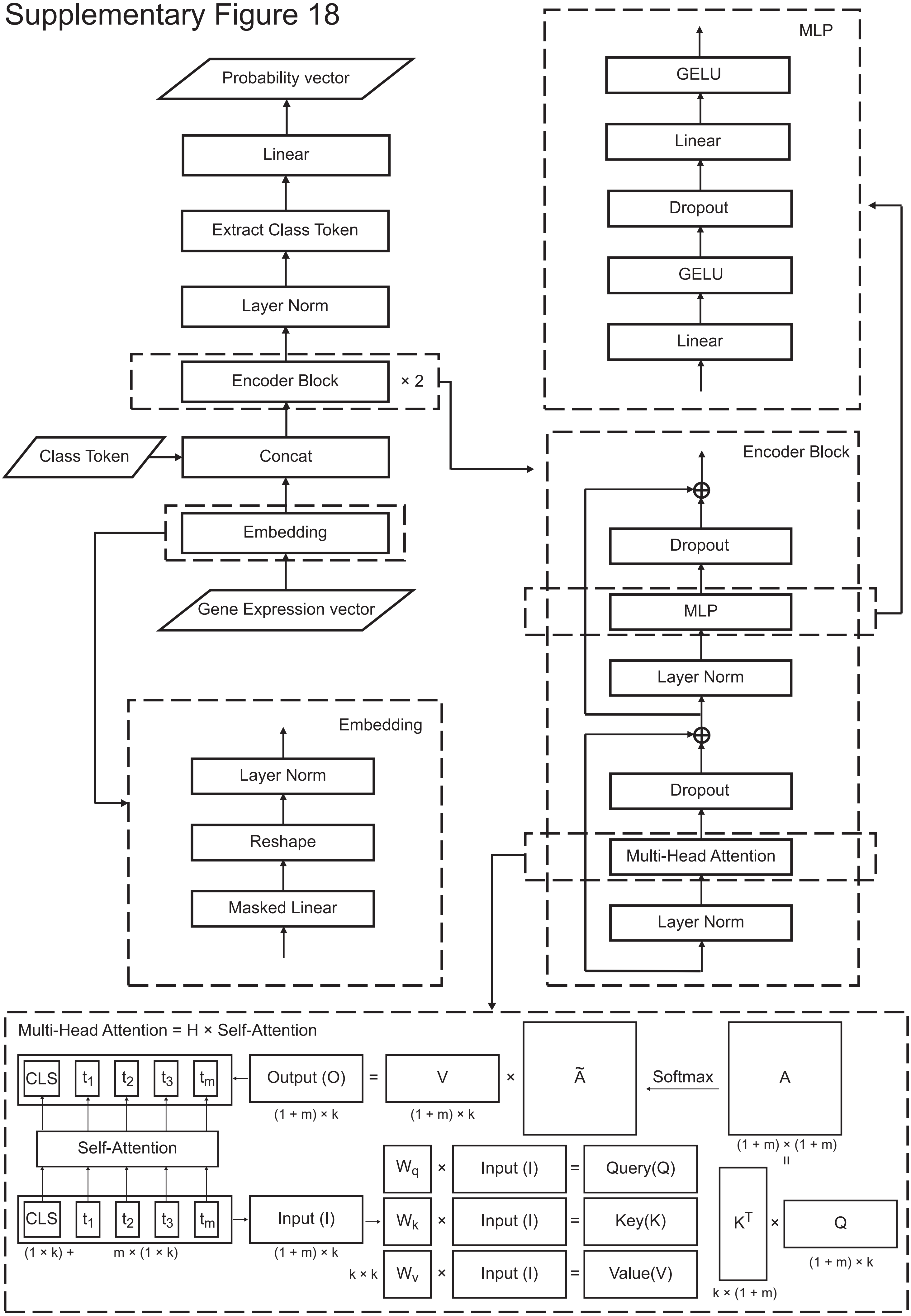

Supplementary Figure 18. Architecture of TOSICA in detail. The gene expression of one cell is first sent into an Embedding layer, and concatenated with class token, then put into Encoder Block which includes the Multi-Head Attention function. Here we use dropout in Encoder Block to avoid of overfitting. Multi-Layer Perception (MLP) is composed of two linear transformations and two GELU activation functions, for learning complex information. Finally, the class token from output of Encoder Block is used as input of a linear classifier. See methods for more details.
